# UBXN2A enhances CHIP‐mediated proteasomal degradation of oncoprotein mortalin‐2 in cancer cells

**DOI:** 10.1002/1878-0261.12372

**Published:** 2018-09-03

**Authors:** Sanam Sane, Andre Hafner, Rekha Srinivasan, Daniall Masood, John l. Slunecka, Collin J. Noldner, Alex D. Hanson, Taylor Kruisselbrink, Xuejun Wang, Yiyang Wang, Jun Yin, Khosrow Rezvani

**Affiliations:** ^1^ Division of Basic Biomedical Sciences Sanford School of Medicine The University of South Dakota Vermillion SD USA; ^2^ Department of Chemistry Center for Diagnostics & Therapeutics Georgia State University Atlanta GA USA

**Keywords:** CHIP E3 ligase, colorectal cancer, mortalin‐2, mouse, UBXN2A, veratridine

## Abstract

Overexpression of oncoproteins is a major cause of treatment failure using current chemotherapeutic drugs. Drug‐induced degradation of oncoproteins is feasible and can improve clinical outcomes in diverse types of cancers. Mortalin‐2 (mot‐2) is a dominant oncoprotein in several tumors, including colorectal cancer (CRC). In addition to inactivating the p53 tumor suppressor protein, mot‐2 enhances tumor cell invasion and migration. Thus, mot‐2 is considered a potential therapeutic target in several cancer types. The current study investigated the biological role of a ubiquitin‐like protein called UBXN2A in the regulation of mot‐2 turnover. An orthogonal ubiquitin transfer technology followed by immunoprecipitation, *in vitro* ubiquitination, and Magnetic Beads TUBE2 pull‐down experiments revealed that UBXN2A promotes carboxyl terminus of the HSP70‐interacting protein (CHIP)‐dependent ubiquitination of mot‐2. We subsequently showed that UBXN2A increases proteasomal degradation of mot‐2. A subcellular compartmentalization experiment revealed that induced UBXN2A decreases the level of mot‐2 and its chaperone partner, HSP60. Pharmacological upregulation of UBXN2A using a small molecule, veratridine (VTD), decreases the level of mot‐2 in cancer cells. Consistent with the *in vitro* results, UBXN2A^+/−^ mice exhibited selective elevation of mot‐2 in colon tissues. An *in vitro* Anti‐K48 TUBE isolation approach showed that recombinant UBXN2A enhances proteasomal degradation of mot‐2 in mouse colon tissues. Finally, we observed enhanced association of CHIP with the UBXN2A‐mot‐2 complex in tumors in an azoxymethane/dextran sulfate sodium‐induced mouse CRC model. The existence of a multiprotein complex containing UBXN2A, CHIP, and mot‐2 suggests a synergistic tumor suppressor activity of UBXN2A and CHIP in mot‐2‐enriched tumors. This finding validates the UBXN2A‐CHIP axis as a novel and potential therapeutic target in CRC.

**Abbreviations**
CHIP: carboxyl terminus of the HSP70‐interacting protein
CRC: colorectal cancer
DMSO: dimethyl sulfoxide
EMT: epithelial–mesenchymal transition
ER: endoplasmic reticulum
HSP: heat‐shock protein
ICC: immunocytochemistry
IP: immunoprecipitation
mot‐2: mortalin‐2
OUT: orthogonal ubiquitin transfer
PBS: phosphate buffer saline
PFA: paraformaldehyde
PFC: prefrontal cortex
RM temperature: room temperature
SE: standard error
TUBE: tandem‐repeated ubiquitin‐binding entities
UBXN2A: ubiquitin‐regulatory X domain N2A
VTD: veratridine
WB: western blot
WT: wild‐type

## 1 Introduction

Colorectal cancer (CRC) is the third leading cause of cancer‐related deaths in the United States. Despite advances in treatment regimens, one‐third of CRC patients will ultimately die from metastatic (disseminated) disease. While the 5‐year survival rate for patients diagnosed with localized CRC is 91%, the prognosis for patients with metastatic disease is an abysmal ≤ 12% (Howlader *et al*., 2016). Understanding the mechanisms of metastatic programs and identifying their negative regulators in CRC cells are, therefore, critical steps in the development of novel therapies that will improve the management of advanced disease. Mot‐2, also called mitochondrial heat‐shock protein 70, heat‐shock protein A9 (HSPA9), or glucose‐regulated protein 75 (GRP75), is a member of the HSP70 chaperone family and has been shown to hold specific tumorigenic roles in different tumors, including CRC (Ando *et al*., 2013; Black and Rezvani, 2016; Cui *et al*., 2017; Dundas *et al*., 2005; Jin *et al*., 2016; Lu *et al*., 2011a; Starenki *et al*., 2015). It has been accepted that mot‐2 overexpression can be a valuable prognostic biomarker as well as a potential target for therapy in patients with breast, brain, liver, and colorectal cancers. Mot‐2's binding protein network allows this oncoprotein to contribute to several steps of tumor development, including sequestration and inactivation of the tumor suppressor protein p53, inhibition of pro‐apoptotic proteins, alteration of the PI3K/AKT signaling pathway, activation of EMT (epithelial–mesenchymal transition), and contribution to cancer cell stemness (Black and Rezvani, 2016; Dundas *et al*., 2005; Gestl and Anne Bottger, 2012; Lu *et al*., 2011b; Na *et al*., 2016; Oki *et al*., 2011; Rozenberg *et al*., 2013; Wadhwa *et al*., 1998, 2006). Inactivation of mot‐2 by siRNA as well as small molecules including withaferin A and MKT‐007 results in growth arrest and induction of apoptosis in cancer cells (Grover *et al*., 2012; Wadhwa *et al*., 2000, 2004).

Our previous findings showed that a UBX (ubiquitin‐regulatory X) domain‐containing protein (Rezvani, 2016) called UBXN2A binds and inhibits mot‐2, resulting in the induction of apoptosis and reduction in cell proliferation in both *in vitro* and *in vivo* models (Abdullah *et al*., 2015b; Sane *et al*., 2014). UBXN2A binds to the substrate‐binding domain of mot‐2 and interferes with mot‐2's protein interaction networks (Sane *et al*., 2016). In addition, we found transcriptional activation of UBXN2A by veratridine (VTD), a plant alkaloid, leads to cell toxicity in a partially mot‐2‐dependent manner (Abdullah *et al*., 2015a).

Heat‐shock proteins are tightly regulated by the ubiquitin–proteasome pathway (Mathew *et al*., 1998). One example is HSP70/HSC70 protein, which engages by the carboxyl terminus of the HSP70‐interacting protein (CHIP) E3 ubiquitin ligase to facilitate the degradation of chaperone clients (Jiang *et al*., 2001; McDonough and Patterson, 2003; Soss *et al*., 2015; Zhang *et al*., 2015). However, upon depletion of misfolded substrates by the HSP70/CHIP complex, CHIP preferentially targets HSP70 and, to a lesser degree, HSC70, for ubiquitination and proteasomal degradation (Qian *et al*., 2006). This sequential ubiquitination of substrates and HSP70 maintains homeostasis by appropriately tuning chaperone levels to reflect the status of protein folding during cell stress as well as the poststress stages. In another previous study, we found that UBXN2A binds CHIP and facilitates its function as an E3 ubiquitin ligase (Teng *et al*., 2015). Based on the above evidence, we hypothesized that UBXN2A as a ubiquitin‐like protein not only binds and interrupts the mot‐2 protein interaction network (Sane *et al*., 2016) but also increases ubiquitination and proteasomal degradation of mot‐2 via the CHIP E3.

Here, we report that UBXN2A facilitates ubiquitination of mot‐2 protein mediated by the ubiquitin‐E3 ligase CHIP in both *in vitro* and *in vivo* models. Induction of UBXN2A promotes ubiquitination and proteasomal degradation of mot‐2 in cancer cell lines in a CHIP‐dependent manner. Using western blotting (WB), flow cytometry, and immunocytochemistry, we show that UBXN2A is required for efficient ubiquitination and degradation of mot‐2 proteins in cancer cell lines and in mouse colon tissues. Silencing UBXN2A in cancer cells with shRNA or haploinsufficiency of UBXN2A expression in UBXN2A^+/−^ mice resulted in an elevation of mot‐2 protein. Pharmacological upregulation of UBXN2A in cancer cells by VTD led to downregulation of mot‐2 in diverse cancer cell lines. Moreover, we found an increased association of CHIP with mot‐2 protein obtained through immunoprecipitation (IP) of UBXN2A from tumors generated by azoxymethane (AOM) and dextran sodium sulfate (DSS) treatment in a C57BL/6 mouse model. Our results uncover a novel regulatory function for UBXN2A that could be essential for the tumor suppressor function of the CHIP E3 ubiquitin ligase previously described in gastrointestinal cancers (Wang *et al*., 2013, 2014a).

## 2 Material and methods

### 2.1 Antibodies, chemicals, and drugs

Primary antibodies and their titers used for WB are summarized in Table S1. Human GST‐tagged mot‐2 recombinant proteins were provided by SignalChem (Richmond, BC, Canada). Recombinant (HIS)_6_‐UBXN2A was produced in BL21(DE3) *Escherichia coli* (BioLabs, Ipswich, MA, USA) using the pRSET C bacterial expression vector as described in the protocol provided by the manufacturer (Thermo Fisher Scientific, Waltham, MA, USA). Human‐UBXN2A was subcloned into the pRSET C vector (Novagen, Madison, WI, USA) to produce a recombinant UBXN2A with a polyhistidine (6xHIS) tag at the N terminus of UBXN2A. We used the magnetic Dynabeads His‐Tag Isolation kit (Thermo Fisher Scientific) for purification of (HIS)_6_‐UBXN2A protein and verified the isolated (HIS)_6_‐UBXN2A with an anti‐His antibody. Veratridine (VTD), an alkaloid extracted from the Veratrum officinale plant, was purchased from Alomone Labs (Jerusalem, Israel). Doxycycline (DOX) was purchased from Clontech (Mountain View, CA, USA). 5‐fluorouracil (5‐FU), etoposide, and emetine were obtained from Sigma‐Aldrich (St. Louis, MO, USA).

### 2.2 Cell culture

Human HCT‐116, LoVo, MCF7, U2OS, HeLa, and HepG2 cancer cells were obtained from the ATCC (American Type Culture Collection, Manassas, VA, USA). All cells were grown in their appropriate mediums, supplemented with 10% fetal bovine serum (Life Technologies, Carlsbad, CA, USA) as well as 100 U·mL^−1^ penicillin and 100 μg·mL^−1^ streptomycin at 37 °C in the presence of 5% CO_2_. HEK293 cells stably expressing scrambled shRNA or shRNA against the CHIP E3 ligase were provided by J. Yin's group. HEK293 cells stably expressing shRNA against CHIP were generated by using GIPZ Human STUB1 shRNA (Clone Id: V2LHS_210715). HEK293 cells were cultured in Eagle's Minimum Essential Medium (ATCC) with 10% FBS and penicillin/streptomycin at 37 °C in a humidified incubator supplied with 5% CO_2_. The tetracycline‐responsive (Tet‐ON) GFP‐UBXN2A and GFP‐empty‐inducible HCT‐116 cells were maintained in McCoy's 5A Medium supplemented with 10% TET‐free FBS, plus penicillin and streptomycin as described above. Appropriate concentrations of puromycin (0.5 μg·mL^−1^) were added to maintain selection of the stably transfected cells after the fourth passage. To induce GFP‐UBXN2A and GFP‐empty expression, DOX (Clontech) was added to the medium. A nontoxic concentration of DOX (2 μg·mL^−1^ media) resulted in efficient induction of the Tet‐dependent promoter (Tilleray *et al*., 2006). To examine the stability of mot‐2 protein ± UBXN2A and CHIP, cells were treated with a protein synthesis blocker, emetine (150 μm), and a proteasome inhibitor, bortezomib (0.5 μm), for 36 and 24 h, respectively. Attached and unattached cells were collected, pelleted on a tabletop centrifuge at 665 ***g*** for 5 min, and washed twice with cold PBS followed by cell lysate preparation and WB analysis. We used GIPZ Human UBXN2A shRNA cloned in lentiviral vectors for silencing UBXN2A. These two clones (Clone ID: V2LHS_212242 and Clone ID: V2LHS_212292) and a scramble clone were provided by Dharmacon (Sane *et al*., 2014). We also used a GIPZ Lentiviral shRNA (Clone ID: V2LHS_210715‐Dharmacon) clone against CHIP. The efficiency of the CHIP shRNA clone was previously confirmed by Yin's Lab. We used a 1 : 1 : 1 ratio of lentiviral shRNAs and GFP‐empty or GFP‐UBXN2A [cloned in pAcGFP1‐C1 (Clontech) plasmids] to transfect cells. We used DharmaFECT kb DNA transfection reagent for triple plasmid transient transfection following the manufacturer's instructions. Cell lysates for WB experiments were prepared using a digitonin lysis buffer [50 mm Tris/HCl, pH 7.5, 150 mm NaCl, 1% digitonin (Sigma‐Aldrich) plus 1× mammalian complete protease inhibitor (Research Products International Corp) (Kisselev and Goldberg, 2005). Cell lysates used in WB and/or immunoprecipitation experiments were normalized for equal loading by a NanoDrop using direct absorbance at 280 nm (Thermo Fisher Scientific).

### 2.3 *In vitro* ubiquitination assay

We set up a ubiquitination assay following previously described methods (Murata *et al*., 2005; Sun *et al*., 2012). Briefly, 2 μg of GST‐mot‐2 was incubated in a 100 μL reaction mixture containing 50 ng of (His)_6_‐UBE1 activating enzyme (E1; Boston Biochem, Cambridge, MA, USA), 0.5 μg of (His)_6_‐UbcH5b Ub‐conjugating enzyme (E2; Enzo Life Sciences, Farmingdale, NY, USA), 2 μg of GST‐CHIP ubiquitin ligase (E3; Ubiquigent, Dundee, Scotland, UK), and 5 μg of GST‐ubiquitin (Boston Biochem). These optimized micrograms of recombinant proteins in the prepared reaction were based on the molecular weights of individual proteins. A reaction buffer (50 mM Tris/HCl, pH 7.5, 4 mM ATP for energy source, and 2 mM MgCl_2_) containing recombinant E1, E2, E3, GST‐ubiquitin, and GST‐mot‐2 received different concentrations of recombinant (His)_6_‐UBXN2A (0, 0.5, 2, and 4 μg) followed by 30 min incubation in a 37 °C water bath. After the *in vitro* ubiquitination assay, the ubiquitinated mot‐2 was pulled down by p62‐UBA (Enzo) immobilized on agarose beads (Rezvani *et al*., 2007, 2009). The UBA domain of p62 is able to bind multi‐ubiquitin chains and pull down ubiquitinated proteins. Yield proteins were subjected to 4–20% gradient SDS/PAGE followed by WB using various antibodies, including an anti‐mot‐2 antibody, to determine the level of polyubiquitinated mot‐2. To exclude the possibility that the GST self‐dimerization may promote artificial ubiquitination of mot‐2 proteins, we used agarose beads containing no immobilized UBA domain as control. The increased high‐molecular ubiquitinated mot‐2 in the presence of UBXN2A and the absence of a typical high‐molecular mot‐2 protein in the control lane excluded the possibility that GST self‐dimerization might have occurred and allowed GST‐ubiquitin to conjugate nonspecifically to its substrates.

### 2.4 Immunoprecipitation, p62 pull‐down assays, and Magnetic Beads TUBE2

Immunoprecipitation (IP) experiments were conducted as previously described using magnetic protein A or protein G beads with immobilized antibodies mixed per the manufacturer's instructions (Thermo Fisher Scientific) (Sane *et al*., 2014). Heavy chains (HC, ~ 55 kDa) and light chains (LC, ~ 25 kDa) of the capture antibody remain present in the eluted IP sample. Yield proteins were subjected to 4–20% gradient SDS/PAGE followed by WB using the appropriate antibodies. A Magnetic Beads TUBE2 (LifeSensors, Malvern, PA, USA) was used to pull down ubiquitinated mot‐protein from cell lysate. The structure of tandem ubiquitin‐binding entities (TUBE) allows magnetic beads to isolate both K48‐ and K63‐polyubiquitinated proteins with equal affinities from cell lysates, allowing for detection at a relatively low abundance of ubiquitinated proteins. Following instructions provided by LifeSensors, polyubiquitinated proteins were separated from magnetic beads for western blotting. The digitonin lysis buffer (Section 2.7) used for the Magnetic Beads TUBE2 experiments additionally contains ubiquitin aldehyde (concentration 2 μm‐LifeSensors). Ubiquitin Aldehyde is a potent and specific inhibitor of deubiquitinating enzymes (DUBs) used to preserve the integrity of polyubiquitin chains on modified proteins for WB. To protect ubiquitinated proteins in treated cells, bortezomib was added to plates 4 h before preparation of cell lysates.

### 2.5 Iodixanol gradient analysis and western blot

We used OptiPrep [60% (w/v) Life Technologies, Inc.], which is a ready‐made solution of Iodixanol. The iodixanol gradient is more efficient in the separation of protein complexes and subcellular compartments because its osmolality and viscosity remain relatively constant with changes in the density of the gradient (Zhang *et al*., 1998). Iodixanol gradient analysis and determination of endoplasmic reticulum (ER), Golgi, and mitochondrial compartments in collected fractions were performed as described (Graham, 2002; Rezvani *et al*., 2009; Sane *et al*., 2014).

Briefly, cell lysates were spun at 13 000 ***g*** for 10 min at 4 °C, and then, supernatants were subjected to a BCA protein assay using a NanoDrop 2000 (Thermo Fisher Scientific). Supernatants were diluted to 2 mg·mL^−1^, and 1 mL of extract was loaded onto a performed iodixanol (v/v) linear gradient (8–38%), as previously described (Sane *et al*., 2014). The gradients were spun at 28 500 r.p.m. (100 000 ***g***) for 18 h using a Beckman SW41Ti rotor at 4 °C (Beckman Coulter Brea, CA, USA). Equal‐volume fractions (~ 0.6 mL) were collected from the top of each gradient and analyzed by WB. For the distribution of major subcellular markers in discontinuous iodixanol gradients, please see references (Rezvani *et al*., 2009; Sane *et al*., 2014).

### 2.6 Immunofluorescence and microscopy

Cells (~ 1 × 10^5^) were seeded onto 18‐mm coverslips in 6‐well plates. Cells received vehicle or treatment (doxycycline or VTD) for the indicated times. Following treatment, cells were further treated in 4% PFA (paraformaldehyde solution) diluted in PBS (phosphate‐buffered saline) for 20 min at room temperature (RT) followed by quenching with 10 mm glycine for 30 s. Permeabilization of cells was achieved with 0.1% NP‐40 in PBS for 5 min at RT. Slides were blocked with 10% donkey serum (Sigma‐Aldrich) for 30 min in RT followed by staining with primary antibodies at 4 °C overnight (mot‐2 antibody: 1 : 400 dilution). Subsequently, cells were washed in RM temperature PBS three times and incubated with fluorescence‐conjugated Alexa Fluor secondary antibodies (Invitrogen; 1 : 500 dilution) for 1 h at RM. Coverslips were mounted with a ProLong Gold antifade reagent with DAPI (Molecular Probes, Eugene, OR, USA) for nuclear staining. Cells were imaged using an Olympus FV‐1000 confocal microscope (Center Valley, PA, USA).

### 2.7 FACS analysis

LoVo and HCT‐116 cancer cells were treated with different concentrations of VTD dissolved in DMSO. After 72 h of treatment, cells were washed twice in ice‐cold PBS, trypsinized, and fixed in 70% ethanol overnight at −20 °C, followed by permeabilization with PBS containing 0.25% Triton X‐100 for 20 min at 4 °C. Then, cells were blocked with 2% BSA in PBS and incubated with anti‐mot‐2 antibody (1 : 100 dilution) per 10^6^ cells for 60 min on ice. Following washing with PBS, cells were incubated with Alexa Fluor 546 donkey anti‐mouse IgG antibody (purple‐Thermo Fisher Scientific, diluted 1 : 50) for 30 min on ice, washed, and resuspended in PBS. Stained cells were analyzed using a bd accuri c6, and the collected data were analyzed using bd accuri c6 analysis software (BD biosciences, Franklin Lakes, NJ, USA).

### 2.8 Cell migration assay using culture inserts

To examine cell migration of the Tet‐on‐inducible HCT‐116 cancer cells, we used an ibidi culture insert system (ibidi). This system has a two‐well silicone insert with a defined cell‐free gap separated by a 500‐mm‐thick wall within a 35‐mm dish. An equal number of GFP‐empty or GFP‐UBXN2A cells were added to the two reservoirs of the same insert and incubated at 37 °C at 5% CO_2_. After 72 h, the insert was gently removed, creating a gap of 500 mm. The plate was filled with complete media, and the migration was photographed using a Leica DMIL microscope (Goyal *et al*., 2011; Henry *et al*., 2015). As previously described (Han *et al*., 2016), we monitored the migration of HCT‐116 at 24 h as well as 48 h after removal of the silicone inserts.

### 2.9 Animals and tissues

Wild‐type (WT) male and female C57BL/6 mice (2 months old) were purchased (from Envigo, Indianapolis, IN, USA). Tissues, including colon tissues, were dissected and homogenized in a digitonin lysis buffer for pull‐down or *in vivo* ubiquitination assay experiments. The UBXN2A‐null mouse line (Ubxn2^atm1(KOMP)Mbp^) was engineered by the Knockout Mouse Project (KOMP, http://www.KOMP.org) in the C57BL/6 background. KOMP used a construct that introduced loxP sites flanking exon 4 so that cell type‐specific KO can be achieved through breeding with a Cre recombinase mouse. Our WB data indicate that UBXN2A(+/−) mice are indeed UBXN2A‐haploinsufficient, as exhibited by a 40–50% reduction (*P* < 0.05) in UBXN2A mRNA and protein in all tissues studied. UBXN2A^−/−^ is embryonic lethal. All animals used, including UBXN2A heterozygous mice (UBXN2A^+/−^) and the WT UBXN2A (UBXN2A^+/+^), were maintained at an animal facility located in the medical school at the University of South Dakota. All procedures were approved by the Institutional Animal Care and Use Committee in accordance with federal guidelines. For *in vivo* ubiquitinated proteins, the lysate buffer had EDTA (5 mm) and deubiquitylases (DUBs) inhibitor N‐ethylmaleimide (10 mm) to preserve proteins in the state of ubiquitination, as previously recommended (Emmerich and Cohen, 2015).

### 2.10 Statistical analysis of data

Unless otherwise stated, at least three biological repeats were performed for all of the cell culture experiments. Statistical values among tested groups were analyzed with either Student's *t*‐test or by one‐way ANOVA followed by Tukey *post hoc* tests, using graphpad prism 7 (La Jolla, CA, USA) when appropriate. The means were compared considering a *P* value of ≤ 0.05 as a significant difference (mean ± SEM) (*P < 0.05, **P < 0.01 and ***P < 0.001).

## 3 Results

### 3.1 Mot‐2 is a substrate for the CHIP E3 ubiquitin ligase

**Figure 1:**
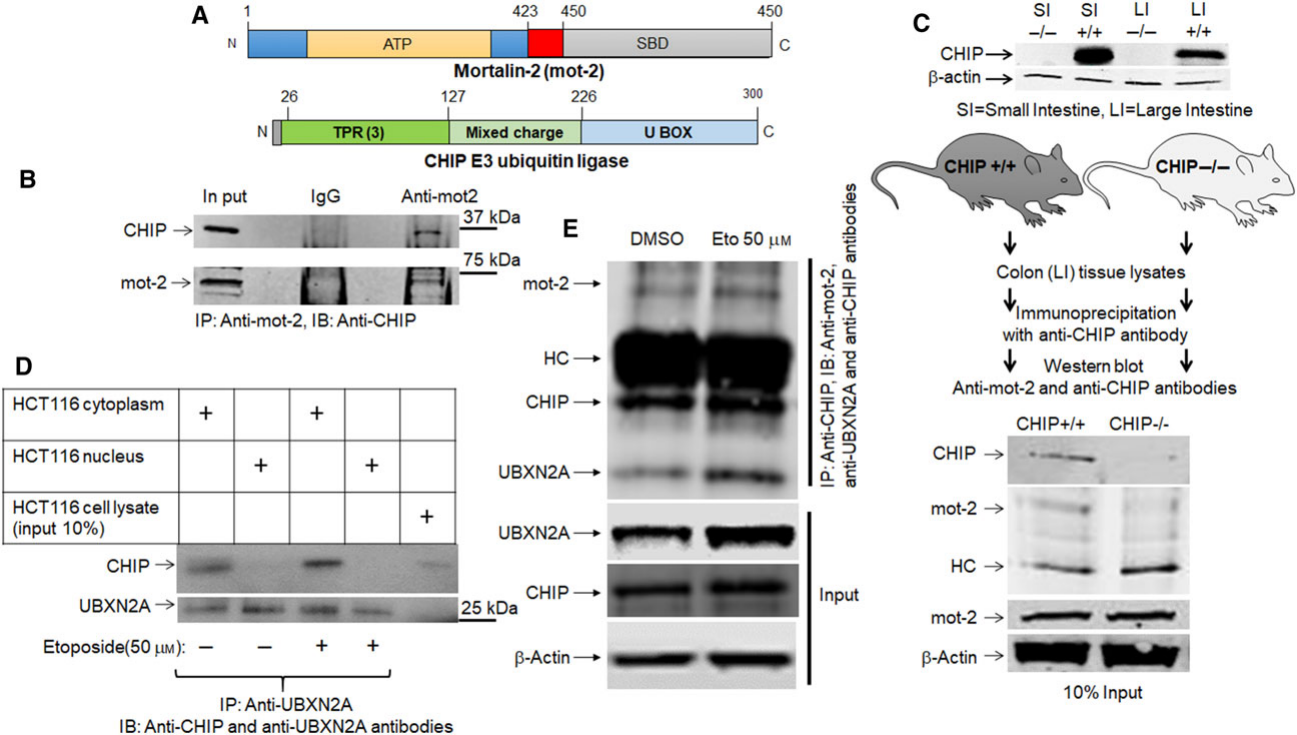
UBXN2A and CHIP proteins form a ternary complex with mot‐2. (A) Schematic drawings of the protein structures of mot‐2 and the CHIP E3 ubiquitin ligase. Mot‐2 has two major domains, an N‐terminal ATPase domain (ATP) and a C‐terminal substrate‐binding domain (SBD). These two domains are reciprocally controlled by the presence of ATP/ADP on the ATP domain and a client protein bound to the SBD (Dores‐Silva *et al*., 2015). The CHIP E3 ubiquitin ligase has three major domains: a tetratricopeptide repeat (TPR) domain located at the N terminus, a U‐box domain at its C terminus, and a mixed‐charge domain located in the middle of the protein (McDonough and Patterson, 2003). (B) HeLa cell lysates were subjected to IP using anti‐mot‐2 antibodies. IP experiments showed mot‐2 can pull down CHIP. (C) WB analysis was used to verify the lack of the CHIP protein in CHIP knockout mice using small intestine (SI) and large intestine (LI) tissue lysates. To verify the mot‐2‐CHIP complex *in vivo*, colon tissue (LI) lysates from C57Bl/6 WT (CHIP^+/+^) or CHIP knockout (CHIP^−/−^) were subjected to IP using anti‐CHIP antibodies immobilized on magnetic IgA. WB showed mot‐2 protein can be pulled down only from WT colon lysates where CHIP proteins are present (HC, heavy chain of immunoglobulins). (D) HCT‐116 cells were exposed to stress using the genotoxic stress agent etoposide for 24 h followed by IP experiments with anti‐UBXN2A antibodies immobilized on magnetic beads coupled with protein A. Control groups received DMSO treatment. WB analysis showed interaction of CHIP and UBXN2A takes place in the cytoplasm and not the nucleus due to the absence of CHIP in the nucleus. More importantly, we observed genotoxic stress induces nucleo‐cytoplasmic translocation of UBXN2A, which leads to an increase in UBXN2A binding to CHIP in cytoplasmic fraction. (E) In another set of experiments, DMSO‐ and etoposide (Eto)‐treated cells were subjected to IP using anti‐CHIP antibodies. WB results show the existence of a triple complex containing mot‐2, CHIP, and UBXN2A. More importantly, E indicates etoposide enhances the association of CHIP and mot‐2 protein alongside an elevation of UBXN2A in the cytoplasm, as previously described. Collectively, the experiments conducted in this figure indicate UBXN2A, mot‐2, and CHIP proteins can be present simultaneously in a multiprotein complex.

We applied the orthogonal ubiquitin transfer (OUT) technique (Zhao *et al*., 2012) to identify the substrate proteins of CHIP E3 ubiquitin ligase in the cell. The OUT system has been established as an efficient platform to identify the direct ubiquitination targets of an E3 enzyme (Liu *et al*., 2017; Wang *et al*., 2017). OUT is enabled by an engineered UB transfer cascade composed of xE1, xE2, and xE3 enzymes that are free of cross‐reactivities with their native partners (‘x’ designates engineered enzymes orthogonal to native enzymes). The xE1‐xE2‐xE3 cascade would exclusively transfer an affinity tagged UB mutant (xUB) to xE3 and then to its substrate proteins in the cell. We previously engineered xUB‐xUba1 and xUba1‐xUbcH5b pairs to enable the exclusive transfer of xUB to xUbcH5b (Zhao *et al*., 2012). We recently used phage display to engineer an xUbcH5b‐xCHIP pair that would deliver xUB to CHIP and then to its substrate proteins (Bhuripanyo *et al*., 2018). We expressed the OUT cascade of CHIP in the HEK293 cells with xUB tagged with 6× Histidine and biotin tags (HBT‐xUB). We used tandem affinity chromatography to purify xUB‐conjugated proteins followed by trypsin digestion and LC‐MS analysis to identify CHIP substrates. One of the candidates from the proteomic screen was mot‐2. Its peptide‐spectrum match (PSM) numbers from cells expressing the full OUT cascade of CHIP are significantly higher than the PSM numbers from control cells expressing xUba1 and xUbcH5b without xCHIP in three independent biological repeats (Table S2). This strongly suggests that CHIP mediates direct transfer of UB to mot‐2. To verify that mot‐2 is indeed a substrate of CHIP (Fig. 1A), we conducted a set of immunoprecipitation experiments using cancer cell lines or colon tissues extracted from CHIP^+/+^ and CHIP^−/−^ mice provided by X. Wang's group (Dai *et al*., 2003; Min *et al*., 2008). Figure 1B shows that CHIP is co‐immunoprecipitated with mot‐2 in HeLa cancer cells. In a reciprocal immunoprecipitation experiment, Fig. 1C shows that CHIP in C57Bl/6 wild‐type pulls down mot‐2 protein in colon tissue extracts, while the mot‐2 pull‐down was unsuccessful in CHIP^−/−^ mice. The input panel in Fig. 1C shows the presence or absence of CHIP has no effect on total levels of mot‐2 in normal colon tissues. This can be due to the absence of endogenous stresses or the dominant mitochondrial localization of mot‐2 in normal cells as previously described (Lu *et al*., 2011b). In addition, the colon tissue lysates used in Fig. 1C were subjected to another set of IP experiments using anti‐UBXN2A antibodies (Fig. S1A). Interestingly, the results showed association of UBXN2A with mot‐2 is enhanced in CHIP^+/+^ mice. However, we observed UBXN2A pulled down some mot‐2 proteins in CHIP^−/−^ mice.

As previously shown (Teng *et al*., 2015) in HEK293 and differentiated PC12 cells, we were able to show that UBXN2A is co‐immunoprecipitated with the CHIP E3 ubiquitin ligase in HCT‐116 colon cancer cells (Fig. 1D). Lane 3 in Fig. 1D shows treatment with a genotoxic agent, etoposide (50 μm), increased association of UBXN2A and CHIP in the cytoplasm due to nuclear–cytoplasmic translocation of UBXN2A in response to etoposide (Abdullah *et al*., 2015b).

Since etoposide could increase levels of UBXN2A and CHIP proteins and also the ratios of UBXN2A vs CHIP following IP experiments, we set up another IP experiment in which we used an anti‐CHIP antibody to pull down CHIP proteins and its associated proteins in the presence and the absence of etoposide. WB analysis showed a triple complex containing mot‐2, CHIP, and UBXN2A in HCT‐116 cell lysates. The IP showed no increase in the level of CHIP proteins with or without etoposide, indicating CHIP is not a target gene for etoposide, as previously investigated (Wang *et al*., 1999). We found that etoposide‐dependent upregulation of UBXN2A in the cytoplasm (Abdullah *et al*., 2015b) can increase association of mot‐2 protein with the CHIP E3 ligase in cytoplasm (Fig. 1E). Altogether, Fig. 1 revealed that CHIP protein binds to mot‐2 oncoprotein in both *ex vivo* and *in vivo* models, verifying the results obtained by the OUT screen. In addition, sustained accumulation of UBXN2A in the cytoplasmic compartment in etoposide‐treated cells enhances CHIP's binding to mot‐2 complexes.

### 3.2 UBXN2A is central to the regulation of mot‐2 stability

**Figure 2:**
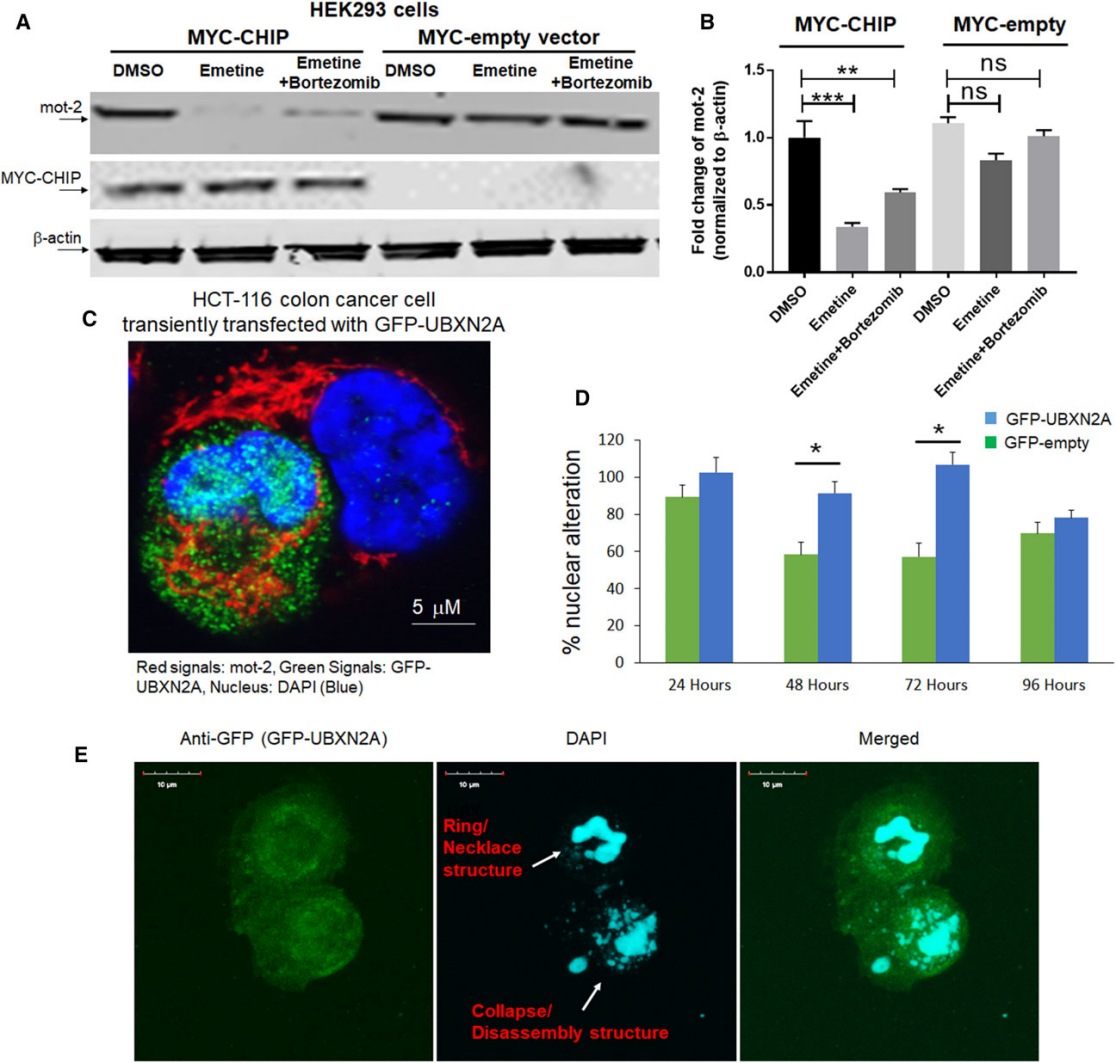
Overexpression of CHIP and UBXN2A decreases mot‐2 protein levels in cells. (A) HEK293 cells were transiently transfected with MYC‐tag CHIP or MYC‐tag empty vectors. After 24 h, cells were treated with DMSO, emetine, or a combination of emetine and bortezomib for another 24 h, followed by WB. (B) Measurement of protein bands revealed overexpression of MYC‐CHIP but not MYC‐empty significantly decreases mot‐2 while in the presence of bortezomib, an inhibitor of the 26S proteasome, downregulation of mot‐2 was blocked in the presence of emetine in cells expressing MYC‐CHIP (*n* = 3, **P < 0.01, ***P < 0.001‐Tukey's multiple comparison test, mean ± SE). (C) HCT‐116 cells were transiently transfected with GFP‐UBXN2A. After 48 h, cells were stained with anti‐mot‐2 followed by fluorescent secondary antibody. (C) z‐stack confocal maximum intensity projection images of mot‐2 (red signals), GFP‐UBXN2A (green signals), and DAPI (blue signals‐nucleus). Scale bar is 5 μm. (D, E) Morphological examination revealed a developing nuclear condensation and fragmentation (Ring/necklace as well as collapse/disassembly structures) in HCT‐116 cells expressing GFP‐UBXN2A in comparison with cells expressing GFP alone in the first 72 h after transient transfection. The number of apoptotic cells started to reduce after 72 h due to formation of resistant cells (*n* = 150 cells per group, *P < 0.05‐ Tukey's multiple comparison test, mean ± SE). Scale bar is 10 μm.

To further confirm CHIP is a dominant E3 ubiquitin ligase for mot‐2 protein, we first used a set of HEK293 cells transiently transfected with empty vector or human CHIP cDNA cloned into a pLenti N‐MYC vector. Twenty‐four hours after transfection, cells were treated with DMSO (0.05%); emetine, a protein synthesis blocker; or a combination of emetine and bortezomib, a selective proteasome inhibitor (Dasgupta *et al*., 2014), for 24 h followed by cell lysates. WB analysis showed that the level of mot‐2 protein was strongly reduced in CHIP‐transfected cells in the presence of emetine while the mot‐2 protein level partially recovered in the presence of the proteasome inhibitor bortezomib (Fig. 2A, lanes 2 and 3 versus lane 1). However, in cells expressing an empty vector, mot‐2 was more stable and decreased to only 80% of the level of DMSO‐treated cells in the presence of emetine (Fig. 2A, lane 5 versus lane 4). As expected, the turnover of mot‐2 was further suppressed in the presence of bortezomib combined with emetine (Fig. 2A, lane 6 versus 4). The bar diagram in Fig. 2B shows the fold change of mot‐2 signals obtained from three independent experiments in duplicate. In a reciprocal experiment, we generated a silencing CHIP cell line by transfecting shRNA into a HEK293 cell line using puromycin selection. HEK293 cells stably expressing scrambled shRNA or shRNA against CHIP were treated with emetine or a combination of emetine and bortezomib for 24 h followed by WB analysis. Results confirmed that silencing CHIP expression increases the protein level of mot‐2 in the presence of emetine. In addition, bortezomib increases stability of mot‐2 protein in the presence of emetine regardless of scramble shRNA or shRNA against CHIP (Fig. S1B). In a set of parallel experiments, we used U2OS osteosarcoma cells stably expressing shRNA against UBXN2A (clones 2 and 3) or scramble shRNA (Sane *et al*., 2016). U2OS cells have an enriched cytoplasmic pool of mot‐2. Figure S1C shows silencing of UBXN2A leads to upregulation of mot‐2, while HSC70 remains intact.

### 3.3 Overexpression of UBXN2A decreases the protein level of mot‐2 and enhances early apoptosis

**Figure 3:**
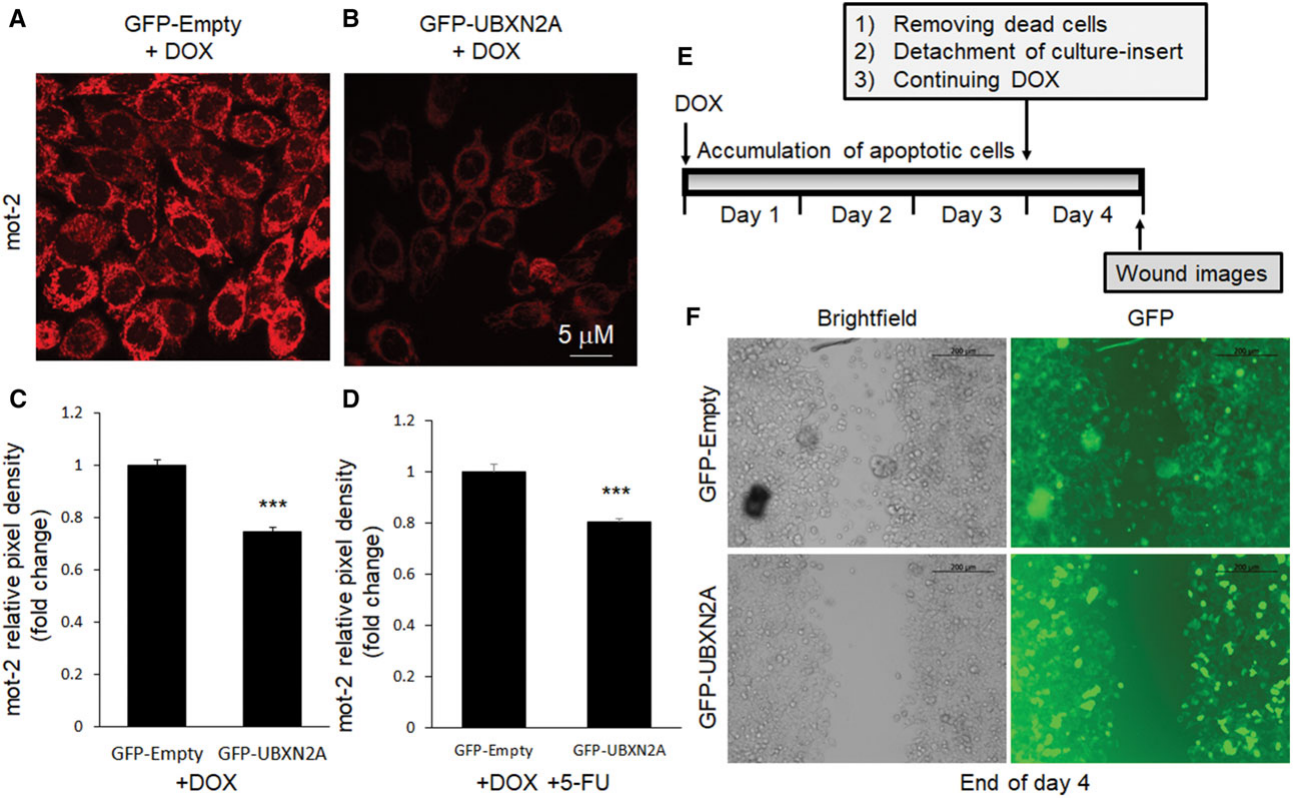
Stable induction of UBXN2A decreases mot‐2 and slows migration in cancer cells developing resistance to apoptosis. We generated a Tet‐on‐inducible HCT‐116 cell line capable of stably expressing GFP‐empty or GFP‐UBXN2A proteins upon DOX treatments. Cells were induced with DOX for 48 h, then fixed and stained for mot‐2 proteins (red signals). (A, B) Representative confocal images of mot‐2 in cells expressing GFP‐empty (A) and GFP‐UBXN2A (B). (C) Quantitative analysis of mot‐2 proteins measured by imagej software (NIH, Bethesda, MD, USA). The mean pixel intensity shows induction of UBXNA leads to significant reduction in mot‐2 by 25% (*n* = 100–150 cells per group, ***P < 0.001 *t*‐test for two groups, mean ± SE). Scale bar is 5 μm. (D) In a similar set of experiments, DOX‐treated cells were simultaneously treated with 5‐fluorouracil (5‐FU), a common antitumor drug for the treatment of human CRC (*n* = 100–150 cells per group, ***P < 0.001 *t*‐test for two groups, mean ± SE). Quantitation of signals indicates that the combination of DOX and 5‐FU has no synergistic suppressive effect on mot‐2 protein levels. (E, F) Tet‐on HCT‐116 metastatic cells were seeded on each side of the Ibidi culture‐insert and treated with DOX for 72 h. Dead/residual cells generated by UBXN2A overexpression were washed out at day 3, and the culture‐insert was detached in order to form a cell‐free gap in the cell monolayer (upper panel). Remaining cells were allowed to migrate for another 24 h. Wound images were obtained using a Leica inverted microscope equipped with a camera. Representative images show more significant closure of the cell‐free gap by cells expressing GFP‐empty than by cells expressing GFP‐UBXN2A after 24 h (F). Scale bar is 200 μm.

In another set of experiments, we used an immunocytochemistry (ICC) technique to analyze the level of stained mot‐2 in the presence and the absence of UBXN2A. HCT‐116 cells were transiently transfected with GFP‐UBXN2A. After 48 h, cultured cells on coverslips were stained with anti‐mot‐2 antibody followed by a 546 Alex Flour fluorescent secondary antibody. The 60% transfection efficiency achieved by Lipofectamine 2000 allowed us to find areas containing transfected and untransfected with GFP‐UBXN2A next to each other. Figure 2C shows two HCT‐116 cells, of which one cell expressed GFP‐UBXN2A while the second cell was untransfected. Comparison of red signals shows GFP‐UBXN2A dramatically decreased the level of mot‐2 (red signals). In addition to the reduction in total red signals, the typical dominant perinuclear localization of mot‐2 (Gao *et al*., 2012) in untransfected cells turned into a filamentous and undiffused aggresome‐like pattern in the presence of GFP‐UBXN2A. As we previously reported, Fig. 2C shows a typical nuclear fragmentation and DNA condensation triggered by UBXN2A in transfected cells versus an intact nucleus in untransfected cells. As previously described (Toné *et al*., 2007), we observed a set of stages of nuclear disassembly starting with ring and necklace condensation in the first 48 h followed by complete nuclear collapse/disassembly after 72 h in transfected cells (Fig. 2D,E). We observed a reduction in DNA fragmentation and chromatin condensation in the remaining transfected cells after 96 h, which may indicate the development of resistant mechanism to the apoptosis program triggered by UBXN2A. A systematic analysis of apoptotic elements and their regulatory factors will be essential to determining the underlying mechanisms behind this cross‐resistance (Saudemont *et al*., 2007), which occurs following initial apoptotic events and cell death regulated by UBXN2A in cancer cells. To further confirm the UBXN2A‐dependent reduction in mot‐2 proteins in cancer cells, we used a Tet‐on system to induce the expression of UBXN2A in HCT‐116 colon cancer cells. Induction of UBXN2A with doxycycline (DOX) for 48 h led to the significant reduction in mot‐2 signals, as compared to cells expressing GFP‐empty vector (Fig. 3A–C). Based on previous report (Lu *et al*., 2011b), we also examined the reduction in mot‐2 protein in UBXN2A‐induced cells in the presence of a chemotherapeutic agent, 5‐Fluorouracil (5‐FU). While the combination of UBXN2A expression induced by DOX and 5‐FU synergistically increased apoptosis events (Sane *et al*., 2016), there was no further reduction in mot‐2 protein levels in combined treatment with DOX and 5‐FU (Fig. 3D). There are two reasons for the unchanged level of mot‐2 when both UBXN2A and 5‐FU are present in cells. Our current preliminary data indicate that UBXN2A targets other signaling pathways besides mot‐2 protein. Therefore, the results presented in Fig. 3D suggest that, while UBXN2A combined with 5‐FU has no further effect on the mot‐2 protein level, they synergistically activate apoptosis in cancer cells. Another possible reason for the absence of change in mot‐2 protein levels could be due to the nature of mot‐2 as a heat‐shock protein. While UBXN2A overexpression decreases mot‐2 protein levels through the ubiquitin–proteasome pathway, mot‐2 can be upregulated transcriptionally in response to genotoxic stress in colon cancer cells (Grimmig *et al*., 2017; Swindell *et al*., 2007) induced by 5‐FU treatment.

Besides its anti‐apoptotic role during the progression of tumors, mot‐2 promotes the epithelial–mesenchymal transition and cancer metastasis (Chen *et al*., 2014; Na *et al*., 2016). Therefore, we decided to examine whether UBXN2A prevents tumor cell migration by down‐regulating mot‐2 after initial pro‐apoptotic effects. Based on the results shown in Fig. 2D, we decided to use our UBXN2A Tet‐on system available in HCT‐116 cells. HCT‐116 cells are known as a high‐metastatic colon cancer cell line (Céspedes *et al*., 2007). HCT‐116 cells seeded on ibidi's dishes were treated with DOX for 72 h and then washed with RT PBS to remove the portion of cells that died upon UBXN2A overexpression (Fig. 3E). The surviving portion of HCT‐116 cells expressing GFP alone or GFP‐UBXN2A were subjected to a cell migration assay using ibidi's culture‐insert system (Section 2) (Goyal *et al*., 2011). While cells expressing GFP alone fully migrated and filled the gap by 24 h, the GFP‐UBXN2A clone showed only minimal migration (Fig. 3F). In another similar set, we allowed cells to migrate for 48 h and we obtained results comparable with the 24 h migration assay.

We also checked the status of the cell cycle at time‐point 0 as well as at 48, 72, and 96 h in treated cells (Fig. S2). Induction of GFP‐UBXN2A induces cell cycle arrest at the G1 phase, analyzed at 48 h. This is likely mediated by the upregulation of p21 previously reported by our group (Abdullah *et al*., 2015a). Interestingly, overexpression of UBXN2A led to elevation of G2/M phase at 72 and 96 h in the remaining cells, which can also lead to cell cycle arrest. There are two reasons for the elevated G2/M phase: (a) It has been reported that the population of cells in the G2/M phase increases upon mortalin knockdown (Wu *et al*., 2013). (b) Longer induction of GFP‐UBXN2A (72 and 96 h) and consequent suppression of p21 can induce cellular senescence phenomena that are represented by elevated G2/M. This p21‐dependent senescence event (Jia *et al*., 2011) along with elevated G2/M is previously reported in resistant human colon cancer cells (Sha *et al*., 2016). In fact, it has been previously reported that changes in cell cycle phases during chemotherapies can determine cells’ response to apoptosis induced by drugs (Beaumont *et al*., 2016). Cancer cells arrested in G2/M can resist apoptosis, suggesting a general escape mechanism that occurs in a heterogeneous population of cancer cells (De Angelis *et al*., 2006; Miwa *et al*., 2015).

The results presented in Figs 2 and 3 plus our previously published data suggest that UBXN2A may have a broad tumor suppressive role in colon cancer cells that extends beyond its direct initiation of apoptotic mechanisms (Sane *et al*., 2014) and targets the mot‐2‐dependent EMT properties of colon cancer cells. The molecular events behind the dual function of UBXN2A in apoptosis and in modulating the epithelial–mesenchymal transition in colon cancer cells are currently under investigation in our group.

### 3.4 UBXN2A promotes ubiquitination of mot‐2 *in vitro*

**Figure 4:**
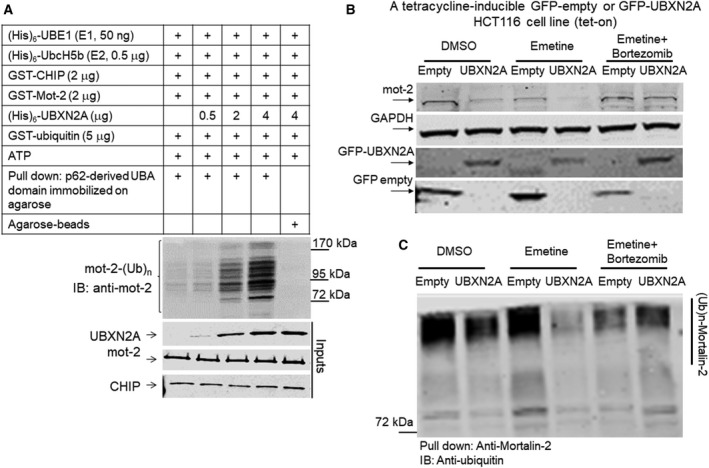
The UBXN2A/CHIP axis promotes ubiquitination and 26S proteasomal degradation of mot‐2 protein. (A) *In vitro* ubiquitination assay containing E1, E2, and CHIP as the E3 ubiquitin ligase, which received recombinant mot‐2 and different concentrations of recombinant UBXN2A. Reactions were subjected to a p62‐UBA pull‐down assay. (A) The typical accumulation of a polyubiquitinated ladder of mot‐2, which was enhanced in the presence of UBXN2A in a dose‐dependent manner. The P62‐UBA strategy followed by immunoblot with anti‐mot‐2 antibodies allowed us to specifically observe ubiquitinated mot‐2 generated in reactions. (B) A set of Tet‐on HCT‐116 cells stably expressing GFP‐empty or GFP‐UBXN2A were treated with DMSO (vehicle), emetine, or a combination of emetine and bortezomib. WB showed UBXN2A decreases mot‐2 protein level while mot‐2 remains intact in the presence of bortezomib regardless of the absence or present of UBXN2A. (C) Cell lysates used in B were subjected to IP using anti‐mot‐2 antibodies immobilized on magnetic beads. WB analysis with FK2 anti‐ubiquitin antibodies revealed UBXN2A decreases the ubiquitinated level of mot‐2 while bortezomib reverses UBXN2A's effect on mot‐2, indicating UBXN2A promotes proteasomal degradation of ubiquitinated mot‐2. Experiments in this figure were repeated two times with similar results.

The presence of the ubiquitin‐regulatory X (UBX) domain, particularly in combination with the ubiquitin‐associated (UBA) domain, allows selective members of the UBXD family to mediate ubiquitination and proteasomal degradation of diverse substrates as part of the ER‐associated degradation (ERAD) mechanism or independent of ERAD (Lee *et al*., 2013; Park *et al*., 2016; Rezvani, 2016; Song *et al*., 2005). For our next step, we decided to determine whether UBXN2A can facilitate/enhance CHIP‐dependent ubiquitination of mot‐2. Figure 4A shows an *in vitro* ubiquitination assay that indicated UBXN2A promotes CHIP‐dependent ubiquitination of mot‐2 in a dose‐dependent manner. A typical polyubiquitinated ladder of mot‐2 was observed particularly in the presence of 4 μg UBXN2A in the reaction. The results shown in Fig. 4A indicate that by increasing UBXN2A, more mot‐2 is ubiquitinated by CHIP. Regulation of a ubiquitin ligase enzyme (CHIP) toward its substrate (mot‐2) by a UBX domain‐containing protein represents a novel paradigm for targeting specific substrates by their known E3 ubiquitin ligases. It is noteworthy that a similar mechanism has been reported for the UBXD7 protein during hypoxia‐inducible factor 1alpha (HIF1α) degradation by the CUL2/VHL E3 ubiquitin ligase (Alexandru *et al*., 2008).

### 3.5 UBXN2A promotes proteasomal degradation of ubiquitinated mot‐2 in colon cancer cells

To confirm whether UBXN2A‐dependent ubiquitination of mot‐2 by CHIP leads to proteasomal degradation of mot‐2 in cells, we used Tet‐on system to control UBXN2A expression in HCT‐116 cells. Figure 4B shows that induction of UBXN2A leads to the downregulation of mot‐2 protein (Fig. 4B, lane 2 versus 1). Reduction in total mot‐2 proteins became more dominant in the presence of emetine in cells expressing UBXN2A (Fig. 4B, lane 4 versus 2). More importantly, we identified that the reduction in mot‐2 protein was blocked in UBXN2A‐expressing HCT‐116 cells treated with both emetine and bortezomib (Fig. 4B, lane 6 versus 4). WB analysis showed that the expression levels of GFP‐empty and GFP‐UBXN2A were similar upon induction by DOX in each Petri dish used for the experiment in Fig. 4B. Our primary experiments indicated that the half‐life of mot‐2 proteins should be around 24 h similar to other HSP70 proteins. We initially prepared cell lysates after 24‐h incubation with emetine since longer incubation with protein synthesis blockers can trigger apoptosis (Lindqvist *et al*., 2012). However, there were inconsistencies between experiments conducted in Fig. 4B. It is noteworthy to say that the presence of emetine additionally triggers an internal stress that can interfere with normal trafficking of mot‐2 between cytoplasm and mitochondria which can also make interpretation of results difficult. We repeated experiments illustrated in Fig. 4B by preparing cell lysate 36 h after adding emetine. We collected all attached and unattached live cells by proper centrifugation for WB analysis (Section 2).

In addition, cell lysates used in Fig. 4B were subjected to IP with an anti‐mot‐2 monoclonal antibody immobilized on IgG magnetic beads. Anti‐ubiquitin WB of the pulled‐down samples showed that UBXN2A can decrease the ubiquitinated level of mot‐2 in cells (Fig. 4C, lane 2 versus 1). The reduction in total levels of ubiquitinated mot‐2 in lanes 2 and 4 indicates that UBXN2A target accumulated ubiquitinated mot‐2 for the proteasomal degradation in the absence of a proteasome inhibitor treatment (Fig. 4C, lanes 4 and 2 versus 3 and 1, respectively), while the levels of ubiquitinated mot‐2 proteins were recovered in the presence of the proteasome inhibitor bortezomib (Fig. 4C, lane 6 versus 4). The FK2 anti‐ubiquitin antibody for WB recognizes polyubiquitinated and monoubiquitinated proteins regardless of linkage location but not free ubiquitin (Veena *et al*., 2014). Collectively, these results indicate that UBXN2A can promote the ubiquitination and proteasomal degradation of mot‐2.

### 3.6 UBXN2A enhances CHIP‐dependent ubiquitination of mot‐2 in cancer cells

**Figure 5:**
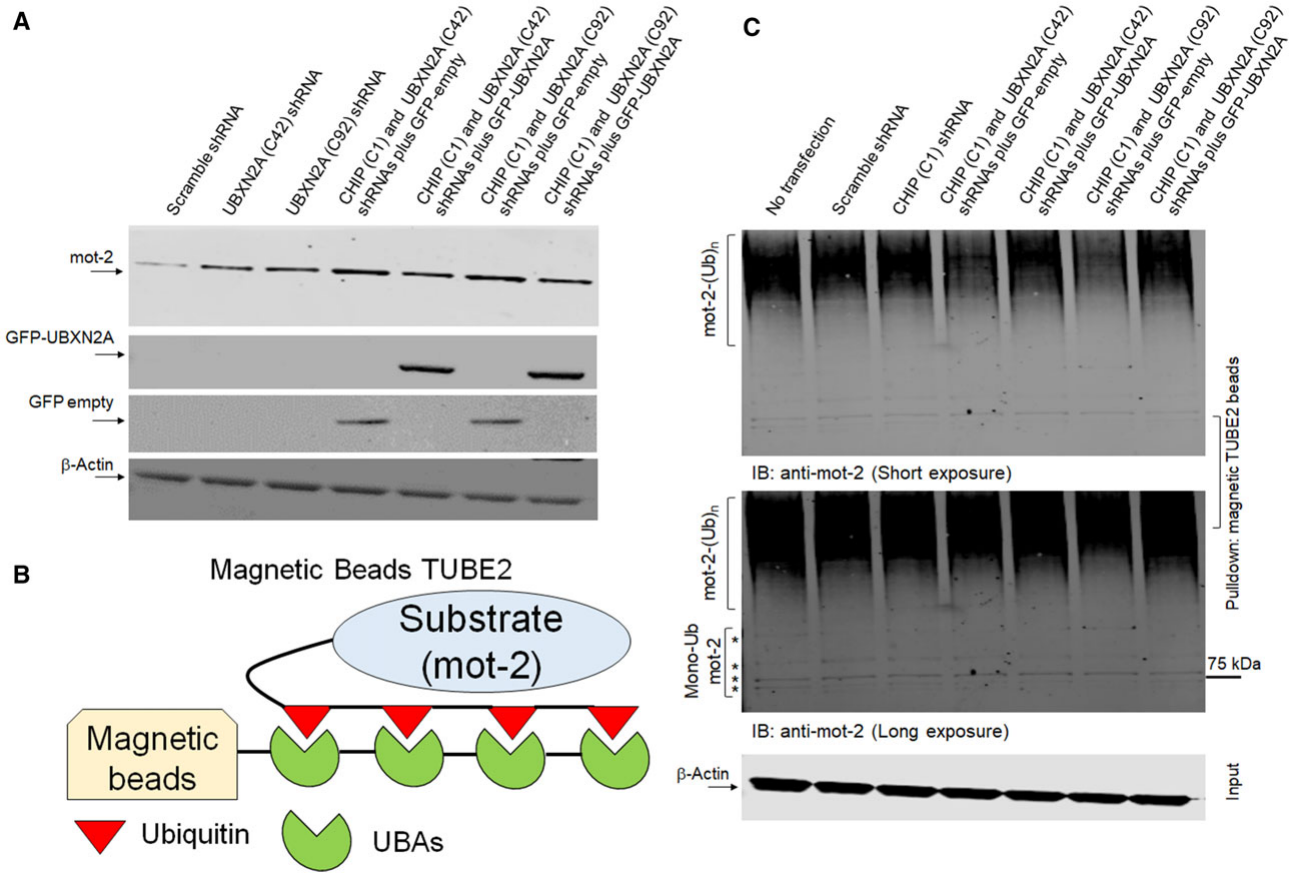
UBXN2A–CHIP axis is essential for ubiquitination of mot‐2. (A, B) HCT‐116 cells were transiently transfected with lentiviral shRNA against CHIP and UBXN2A as well as GFP‐empty or GFP‐UBXN2A plasmids. (A) Western blot analysis indicates increased mot‐2 protein in cell expressing shRNA against UBXN2A alone and an even further increase when both UBXN2A and CHIP shRNA are present (lanes 2, 3, 4, and 6 versus lane 1). However, re‐expression of GFP‐UBXN2A and not GFP‐empty in UBXN2A‐silenced cells led to a reduction in mot‐2 protein (lanes 5 and 7 versus lanes 4 and 6). (B, C) Cell lysates were incubated with Magnetic Beads TUBE2 followed by WB analysis. WB showed a combination of UBXN2A and CHIP shRNAs decreases the ubiquitinated mot‐2 proteins (lanes 4 and 6 versus lane 1) while re‐expression of GFP‐UBXN2A and not GFP‐empty increases the ubiquitinated mot‐2 proteins (lanes 5 and 7 versus lanes 4 and 6). Longer exposure showed no major changes in mono‐ and multi‐monoubiquitination mot‐2 proteins expressing shRNAs and GFP‐empty or GFP‐UBXN2A plasmids. Bortezomib was added 4 h before preparing cell lysates. An aliquot of total cell lysates (20% of IP) was used as the input control.

To understand the impact of UBXN2A and CHIP on mot‐2's ubiquitination and turnover, we first silenced UBXN2A using two efficient lentiviral shRNAs (Sane *et al*., 2016). Lanes 2 and 3 in Fig. 5A show the absence of UBXN2A increases the protein levels of mot‐2. In the next set of experiments, cells were simultaneously transfected with shRNA against UBXN2A (clones 42 and 92) and CHIP (clone 1) together with GFP‐empty or GFP‐UBXN2A (Section 2). Comparison of lanes 4 and 6 (expressing shRNAs and GFP‐empty) to lanes 5 and 7 (expressing shRNAs and GFP‐UBXN2A) in Fig. 5A indicate that re‐expression of UBXN2A can decrease the extent of Mot‐2 elevation due to the silencing of UBXN2A and CHIP by shRNAs. In the next step, we employed a Magnetic Beads TUBE2 (Fig. 5B) to pull down ubiquitinated mot‐2 protein in cells expressing shRNA against CHIP and UBXN2A plus GFP‐empty or GFP‐UBXN2A. Cells used in Fig. 5C were treated with bortezomib before preparing cell lysates (Section 2). WB experiments showed that silencing of the CHIP E3 ligase has no significant effects on the ubiquitinated level of mot‐2 cells (lane 3 versus lanes 1 and 2). However, in triple transfection (lanes 4–7), we observed significant reduction in mot‐2 in the absence of both UBXN2A and CHIP (lanes 4 and 6 versus lane 2). More importantly, re‐expression of GFP‐UBXN2A reverses the effect of CHIP and UBXN2A shRNAs by increasing ubiquitinated mot‐2 (lanes 5 and 7 versus 4 and 6). A longer exposure (Fig. 5C, middle panel) showed less significant changes in mono‐ and poly‐monoubiquitinated mot‐2 proteins with and without GFP‐UBXN2A (see stars in middle panel). To show mot‐2 is a specific substrate for CHIP‐UBXN2A complex, we probed the same membrane with anti‐ubiquitin antibody (Clone FK2). Panel A in Fig. S3 shows shRNAs against UBXN2A and CHIP ± GFP‐UBXN2A have no effect on the total levels of ubiquitinated proteins. Altogether, these data indicate UBXN2A can promote mot‐2 ubiquitination and degradation via the E3 ligase CHIP.

### 3.7 UBXN2A inducer VTD increases proteasomal degradation of mot‐2 proteins in colon cancer cells

**Figure 6:**
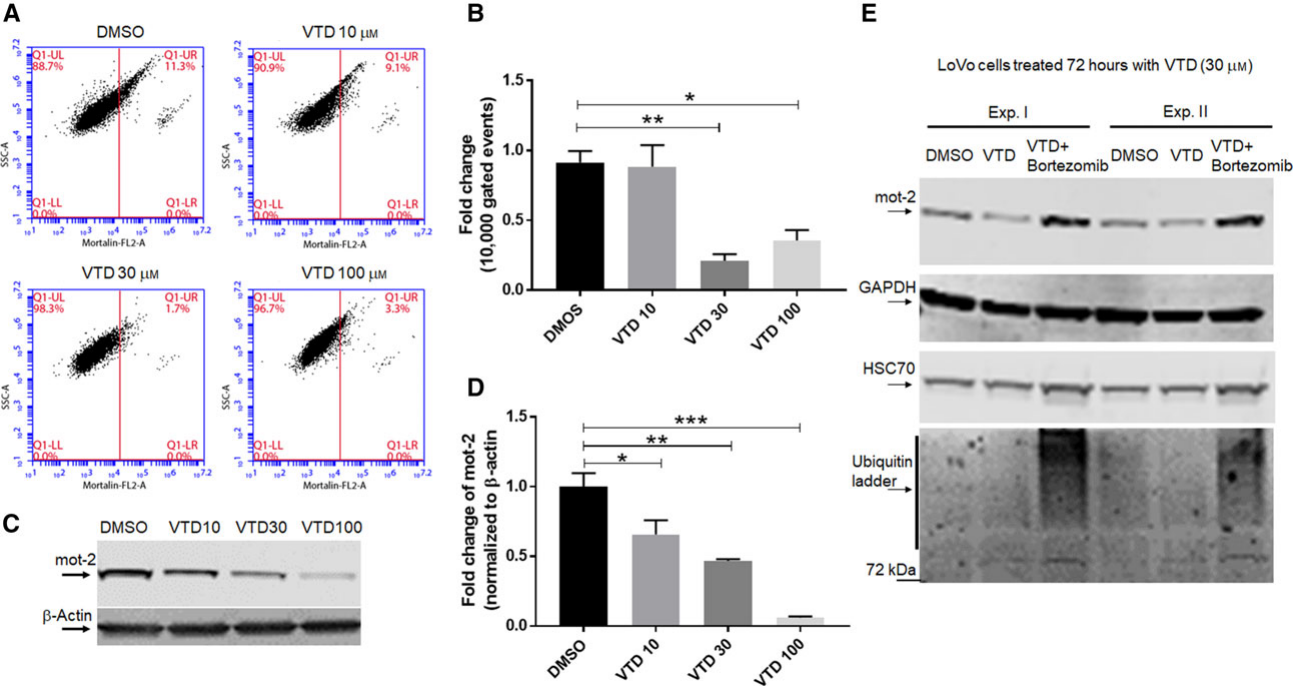
LoVo cells treated with VTD show a dose‐dependent reduction in mot‐2 levels. (A, B) LoVo colon cancer cells were treated with the indicated concentrations of VTD or vehicle (DMSO) for 72 h followed by staining with anti‐mot‐2/Alexa Fluor 546. Cells were examined using flow cytometry, and data were normalized with DMSO. Data are shown as mean ± SEM of three data readings (*n* = 3) where *P < 0.05 and **P < 0.01 using Tukey's modified Student's *t*‐test. (C, D) In a similar set of experiments, LoVo cells were treated with DMSO or different concentrations of VTD for 72 h. Cell lysates were subjected to WB experiments. Statistical analysis of mot‐2 protein bands normalized by β‐actin shows VTD significantly deceases mot‐2 proteins in a dose‐dependent manner (*n* = 3, *P < 0.05, **P < 0.01, ***P < 0.001 Tukey's modified student's *t*‐test, mean ± SEM). (E) Three groups of LoVo cells were treated with DMSO, VTD (30 μm), or VTD (30 μm) plus bortezomib for 72 h, followed by cell lysate preparation. Bortezomib was added 24 h before preparing cell lysates. WB analysis showed VTD‐dependent reduction in mot‐2 can be reversed by bortezomib, while VTD showed no effects on HSC70. Lower Panel E shows the efficient accumulation of ubiquitinated proteins in the presence of bortezomib. Experiments in Panel E were repeated two times with similar results.

A high‐throughput drug screen identified the plant alkaloid VTD as a potent inducer of UBXN2A transcription. VTD promotes cell death in CRC cells in a UBXN2A‐dependent manner and acts synergistically with chemotherapeutic agents (e.g., 5‐fluorouracil) (Abdullah *et al*., 2015a; Sane *et al*., 2016). We decided to determine whether VTD‐dependent overexpression of UBXN2A leads to downregulation of mot‐2 proteins in colon cancer cells. As previously shown (Abdullah *et al*., 2015a), we first reconfirmed that VTD enhances expression of UBXN2A in a dose‐dependent manner in LoVo (Fig. S3B,C), a well‐differentiated colon cancer cell line. LoVo cells were treated with different concentrations of VTD for 72 h. After 72 h, cells were fixed and permeabilized, and mot‐2 proteins were stained by anti‐mot‐2 antibody (1 : 100 dilution) followed by binding to fluorescently conjugated secondary antibodies (Alexa Fluor 546, 1 : 50 dilution). Stained cells were analyzed by flow cytometry for red fluorescence using a laser emission of 560–640 nm (FL‐2). Figure 6A shows representative diagrams of flow cytometry analysis of stained mot‐2 in the LoVo cancer cell line after gating out dead cells, cell debris, and doublets. Compensation for background fluorescence was performed by including target signals of only the secondary antibody (negative control) and cells treated with DMSO. Figure 6B shows the average percentages from three individual analyses performed in triplicate. Panels A and B in Fig. 6 show that VTD‐dependent overexpression of UBXN2A leads to significant downregulation of mot‐2 proteins at 30 μm as well as 100 μm VTD. In the second set of experiments, LoVo cells treated with different concentrations of VTD for 72 h were subjected to WB analysis (Fig. 6C). Image studio (version V) was used to compare the intensity of gray‐scale bands on visualized nitrocellulose membrane by a LI‐COR Odyssey infrared imager (Lincoln, NE, USA). After normalization with β‐actin (the housekeeping gene), statistical analysis showed that VTD‐dependent overexpression of UBXN2A significantly decreases the protein levels of mot‐2 in a dose‐dependent manner (Fig. 6D). In a separate set of experiments, we incubated LoVo cells with 30 μm VTD for 72 h. One set of plates additionally received bortezomib for the final 24 h. Figure 6E shows two independent WB experiments in the presence of DMSO, VTD 30 μm, or a combination of VTD 30 μm plus bortezomib. The results show that UBXN2A induced by VTD reduces the protein level of mot‐2 proteins, while the presence of a proteasome inhibitor, bortezomib, blocks the reduction in mot‐2 triggered by VTD. Interestingly, probing membranes with anti‐HSC70 monoclonal antibody revealed no changes in the presence or the absence of VTD, while HSC70 showed an increase in the presence of bortezomib, as previously described (Kundrat and Regan, 2010; Qian *et al*., 2006). The lower panel in Fig. 6E shows the accumulation of ubiquitinated proteins in lanes 3 and 6, which confirms that bortezomib efficiently inhibited the 26S proteasome complexes in LoVo cells. In another set of experiments, we used HEK‐293 cells stably expressing shRNA against CHIP, which were transfected with UBXN2A shRNA lentiviral vectors (clones 42 and 92). Two days after transfection, cells were treated with DMSO or VTD (100 μm) for another 72 h. Figure S3D shows VTD failed to decrease the protein level of mot‐2 in the absence of UBX2A and CHIP (lane VI versus V and lane III versus II). This result indicates VTD works directly through CHIP and UBXN2A to destabilize mot‐2 in cancer cells. In addition, the VTD/UBXN2A axis selectively targets mot‐2 proteins, while other members of the heat‐shock protein family, such as HSC70, can remain intact in VTD‐treated cells. Figure S3D shows VTD induced UBXN2A expression decreases mot‐2 levels even when CHIP is silenced. It is possible that the CHIP silencing was not complete or other E3s pairing with UBXN2A in VTD‐treated cells can still assist UBXN2A‐dependent ubiquitination of mot‐2.

Immunofluorescent (IF) studies further confirmed that VTD can indeed lead to reduction in total mot‐2 proteins in LoVo cells (Fig. S4). To show that VTD‐dependent reduction in mot‐2 does not only occur in well‐differentiated colon cancer cells, we repeated the VTD experiments in HCT‐116 cells, a highly metastatic colon cancer cell line (Li and Chen, 2010). Flow‐cytometry and WB studies in Fig. S5 show that the VTD/UBXN2A axis can significantly decrease mot‐2 proteins in a dose‐dependent manner. The significant reduction in mot‐2 in the presence of a low concentration of VTD (10 μm) measured by the fluorescence‐activated cell sorting (FACS) technique shows VTD‐dependent reduction in mot‐2 may be more effective in metastatic colon cancer cells. This may be due to a low level of basal UBXN2A in metastatic cells dramatically triggered by VTD (Abdullah *et al*., 2015a). In addition, the dominant anti‐mot‐2 effect of VTD observed in metastatic cells such as HCT‐116 might be due to the endogenous stresses present in metastatic cells, as previously described (Lu *et al*., 2011b). In addition to colon cancer cells, HepG2, a human liver hepatocellular carcinoma cell line, and MCF‐7, a human breast cancer cell line, were treated with different concentrations of VTD for 72 h followed by IF studies. The results in Fig. S6 show that VTD decreases the level of mot‐2 proteins in HepG2 and MCF‐7 cells in variable degrees. Together, these results indicate that VTD can decrease the protein levels of mot‐2 via upregulation of UBXN2A in a dose‐dependent manner in diverse cancer types. However, VTD‐dependent reduction in mot‐2 proteins is variable and determined by the genetic and epigenetic background of the examined cells.

### 3.8 VTD reduces mot‐2/HSP60 chaperone complexes in both cytoplasm and mitochondrial compartments

**Figure 7:**
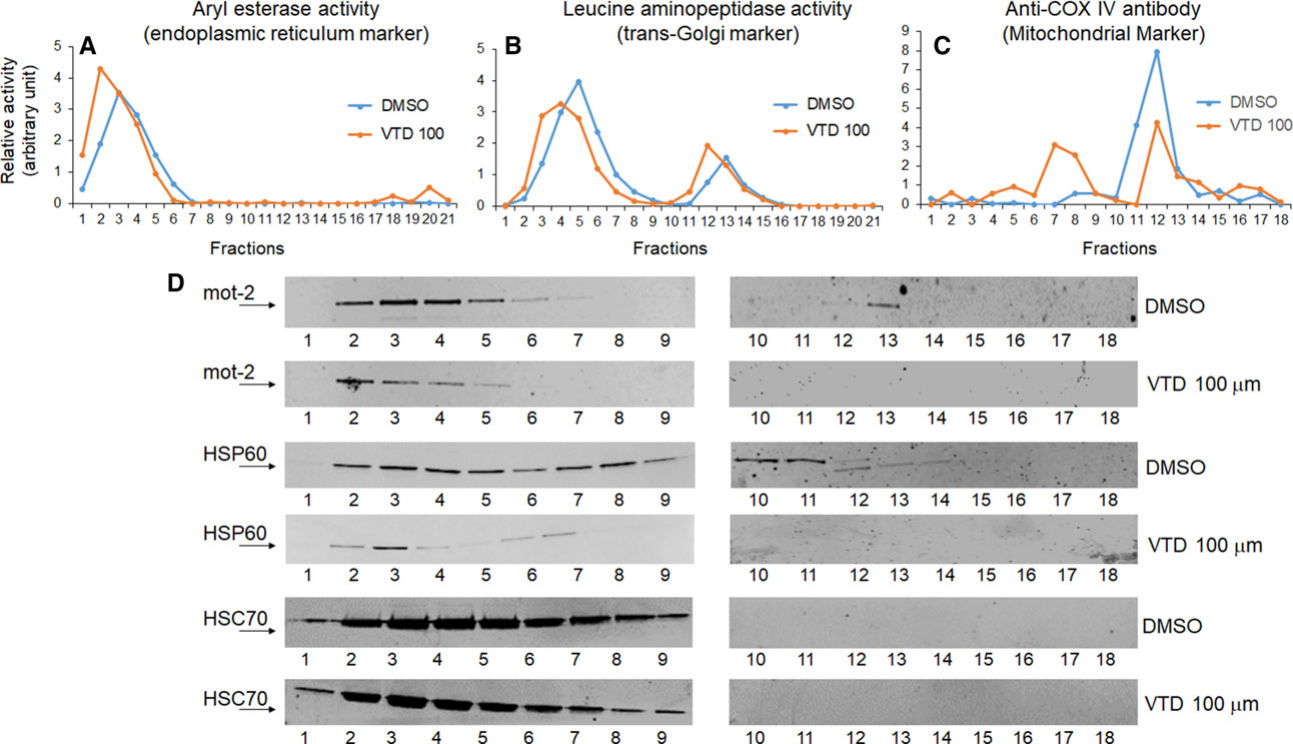
VTD selectively decreases protein levels of mot‐2 and its chaperone partner, HSP60, in both cytoplasmic and mitochondrial compartments. LoVo cells were treated with 100 μm VTD for 72 h followed by iodixanol gradient ultracentrifuge fractionations. (A–C) ER, Golgi, and mitochondrial markers determined the locations of these three membrane compartments. (D) Collected fractions (1–18) were probed with anti‐mot‐2, anti‐HSP60, and anti‐HSC70 antibodies. Results show a dramatic reduction in mot‐2 and HSP60 in both cytosolic‐ and mitochondrial‐enriched fractions, whereas HSC70 levels slightly decreased in the presence of VTD.

Besides endoplasmic reticulum (ER) and cytoplasmic localization (Gestl and Anne Bottger, 2012; Takano *et al*., 2001), a 46‐amino acid‐long mitochondrial‐targeting signal peptide guides the mot‐2 precursor to the mitochondrial matrix. Mot‐2 binds to incoming segments of unfolded preproteins and facilitates their translocation across the mitochondrial membranes using ATP (adenosine triphosphate) as a source of energy (Deocaris *et al*., 2006; Schneider *et al*., 1994). Mot‐2‐dependent internalization of nuclear‐coded proteins to mitochondria is a necessary step for importing cytosolic proteins before they go through folding and protein quality control steps dominantly maintained by mot‐2's chaperone partner, HSP60, in the mitochondrial matrix (Agsteribbe *et al*., 1993; Deocaris *et al*., 2008; Langer and Neupert, 1991). In fact, current evidence indicates that mot‐2 and HSP60 work in coordination to properly maintain protein import and folding processes in mitochondria, and the absence of each of these two proteins interferes with the normal function of mitochondria and, consequently, the growth arrest of cancer cells (Wadhwa *et al*., 2005). Previous evidence shows that not only does mot‐2 colocalize with HSP60, but also these two proteins are affected in a parallel fashion in response to other cell events and stimulants (Bruschi *et al*., 1993; Wadhwa *et al*., 2005). Based on this evidence, we decided to determine whether UBXN2A‐dependent degradation of mot‐2 can consequently lead to a reduction in mot‐2's protein partner, HSP60. We utilized an iodixanol gradient fractionation method to separate cellular compartments. This approach allows satisfactory isolation of the ER, Golgi, and mitochondrial membrane compartments. LoVo cells were treated with DMSO or 100 μm VTD for 72 h followed by an iodixanol gradient fractionation (Section 2). Using substrates specific for ER and Golgi compartments and anti‐COX IV antibody, we first determined the locations of these subcellular compartments in collected fractions (Fig. 7A–C). WB studies showed that VTD‐dependent overexpression of UBXN2A decreases mot‐2 levels in both cytoplasmic fractions, those associated with ER and Golgi compartments as well as mot‐2 proteins cofractionated with the mitochondrial compartment (fractions 10–14) (Fig. 7D). Furthermore, WB in Fig. 7D revealed that HSP60, a major partner of mot‐2, shows a similar reduction in both cytoplasmic and mitochondrial compartments alongside reduction in mot‐2 proteins. On the other hand, the level of HSC70 showed no changes in response to VTD (Fig. 7D, the last two lower panels). Taken together, these results indicate that VTD‐dependent overexpression of UBXN2A decreases the protein level of mot‐2 and consequently its ‘chaperone partner’ HSP60. Solid evidence in the literature has elucidated the dominant role of mot‐2 and HSP60 in cancer cell growth through mitochondrial protein homeostasis (Wadhwa *et al*., 2005) as well as their roles in cancer transformation (Czarnecka *et al*., 2006) and apoptosis (Ghosh *et al*., 2008; Yoo *et al*., 2010). Previously reported growth arrest and apoptosis events induced by VTD (Abdullah *et al*., 2015a) can be partly intermediated through the simultaneous inhibition of mot‐2 and HSP60 proteins by the VTD‐UBXN2A axis in cancer cells. Further studies are required to determine whether VTD‐UBXN2A primarily targets cytoplasmic mot‐2 for ubiquitination by the CHIP E3 ubiquitin ligase and whether the reduction in mot‐2 and HSP60 in mitochondrial compartments is a secondary response to the reduced cytoplasmic pool of mot‐2.

### 3.9 UBXN2A modulates ubiquitinated mot‐2 proteins in mouse colon tissues

**Figure 8:**
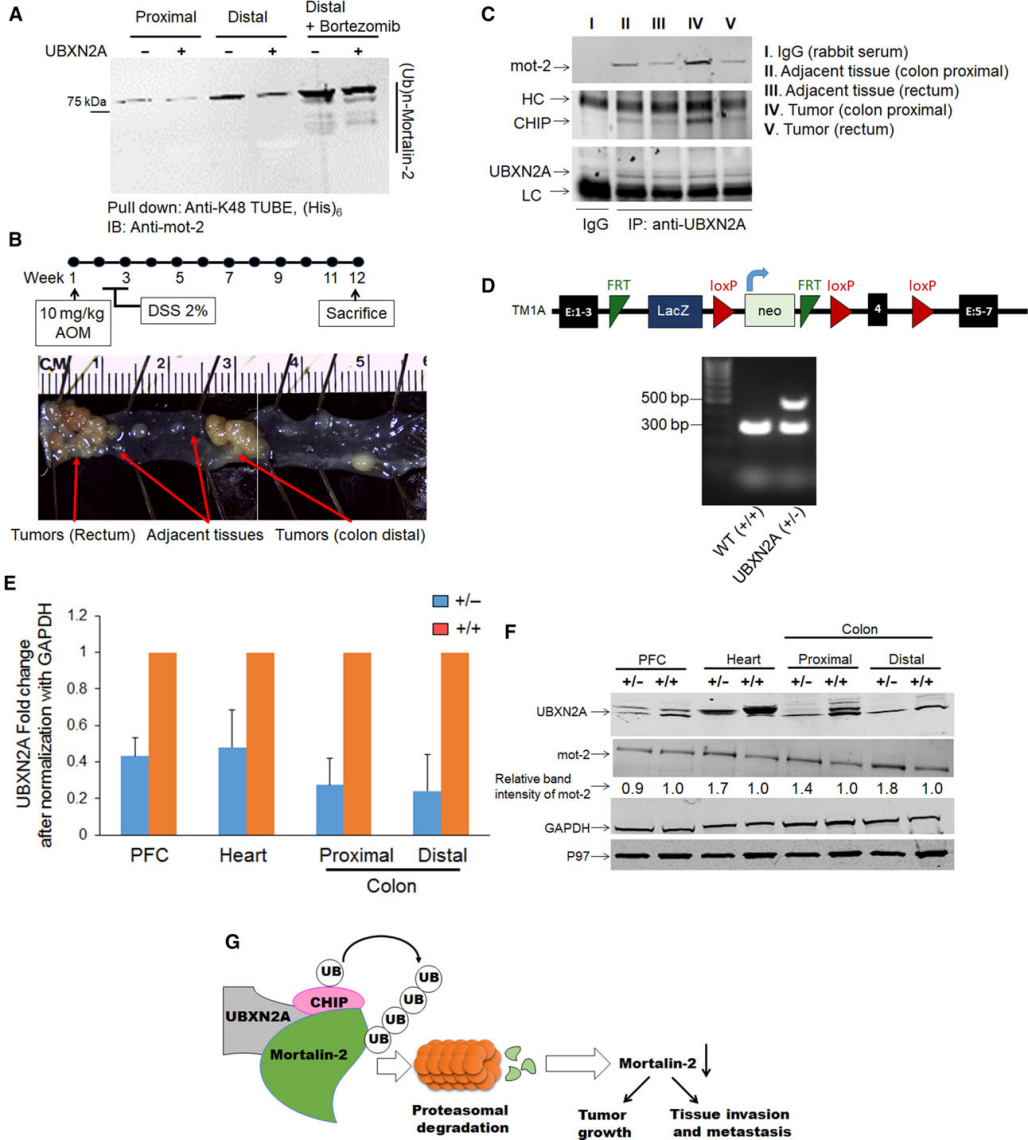
UBXN2A/CHIP is associated with mot‐2 in colorectal tissues and targets endogenous mot‐2 for proteasomal degradation in colorectal tumors. Whole tissue homogenates contain all enzymatic reactions for proper function of the endogenous ubiquitin–proteasome pathway, and therefore, they can be an ideal starting material for examining the *in vivo* proteasomal degradation of mot‐2 in the presence of recombinant UBXN2A proteins. (A) Tissue homogenates in the presence and the absence of GST‐UBXN2A were subjected to pull‐down experiments using K48‐TUBE followed by WB using anti‐mot‐2 antibodies. In addition, a set of tissue homogenates were additionally treated with bortezomib, a potent 26S proteasome inhibitor. (B) Normal adjacent tissues and colorectal tumors were dissected from the AOM/DSS colon cancer mouse model. Normal adjacent and tumor tissues were pooled from three animals for IP experiments. (C) Tissue lysates were subjected to IP using anti‐UBXN2A antibodies immobilized on IgA magnetic beads. Pulled‐down proteins were subjected to WB experiments using anti‐mot‐2, anti‐CHIP, and anti‐UBXN2A antibodies [I and II. Adjacent tissues (colon Distal); III. Adjacent tissues (rectum); IV. Tumors (colon Distal); V. Tumors (rectum)]. Results indicate association of UBXN2A with mot‐2 and the CHIP E3 ubiquitin ligase, particularly in tumors originated in the colon area. Experiments illustrated in A and C were repeated three times with similar results (HC: heavy chain and LC: light chain). (D) Schematic diagram of UBXN2A^tm1a(KOMP)Mbp^ allele. The UBXN2A tm1a allele was initially a nonexpressive form due to the trapping cassette (LacZ/Neo), disrupting the UBXN2A transcript. Lower panel in D shows an agarose gel image of PCR products for WT (301 bp) and heterozygous UBXN2A (+/−, 301 bp and 514 bp) mice (*n* = 3). The left lane (MW) shows DNA molecular weight markers. (E, F) Western blotting of PFC, heart, and proximal and distal colon tissues dissected from WT and UBXN2A heterozygote (UBXN2A^+/−^) mice revealed haploinsufficiency of UBXN2A (E), (*n* = 4 per group error bars display SE to an observed mean in +/− mice group) can lead to elevation of mot‐2, particularly in distal colon tissues. Numbers shown below mot‐2 protein bands are relative intensities of the bands with the level in WT tissues as 1.0. Western blots shown are representative of three independent experiments (F). UBXN2A haploinsufficient mice display a moderate reduction in UBXN2A's protein partner, p97 as well. (G) UBXN2A facilitates ubiquitination of mot‐2 oncoprotein by the CHIP E3 ubiquitin ligase in cancer cells.

To verify whether UBXN2A can target mot‐2 in colon tissues for proteasomal degradation similar to colon cancer cells, we prepared tissue homogenates of proximal and distal colon from C57Bl/6 mice. Equal amounts of tissue lysates were incubated for 1 h at 37 °C with gentle rocking, with and without 0.5 μg recombinant GST‐tag human UBXN2A protein. In addition to recombinant UBXN2A, a separate set of tissue lysates received bortezomib (0.5 μm). In the next step, all six reactions were incubated with HIS K48‐TUBE for 1 h on ice followed by magnetic HIS pull‐down, according to the manufacturer's instructions (LifeSensors). Ubiquitinated mot‐2 bound to K48‐TUBE (Hjerpe *et al*., 2009) was subjected to WB using anti‐mot‐2 monoclonal antibody. Figure 8A shows that the presence of UBXN2A decreases the ubiquitinated level of mot‐2 in both proximal and distal colon tissues. It is noteworthy that the ubiquitinated mot‐2 dominantly appeared as a single ubiquitinated mot‐2 band, as previously reported (Bertrand *et al*., 2014); see also Fig. S7A,B. More importantly, Fig. 8A shows that the present of bortezomib stabilized the level of ubiquitinated mot‐2 regardless of the absence or presence of recombinant UBXN2A. More interestingly, we observed a more typical ladder of ubiquitinated mot‐2 in the presence of bortezomib (lanes 5 and 6). The protein marker in the Fig. 8A panel shows some of the bands that reacted with anti‐mot‐2 antibodies are below 75 kDa in lanes 5 and 6. These bands may represent truncated forms of mot‐2 proteins that carry a mono‐ubiquitin or ubiquitin chains, and therefore, they were pulled down by the HIS K48‐TUBE system. Generation of truncated substrates due to partial proteolysis by the proteasome complex has been previously described, particularly for regulatory and chaperone proteins. Collectively, the data indicate that UBXN2A participates in the ubiquitination process of mot‐2 protein both *in vitro and in vivo*.

### 3.10 A multiprotein complex contains UBXN2A‐CHIP‐mot‐2 predominantly present in colorectal tumors

During the progression of tumors, multiple changes occur in the chaperone/cochaperone complexes that subsequently alter patients’ response to treatment and impact patient survival (Black and Rezvani, 2016; Melle *et al*., 2006; Ruckova *et al*., 2012). CHIP, as part of the HSP70 complex, performs different functions in various human cancers, including a tumor suppressor function in colorectal, breast, prostate, and gastric cancers (Blessing *et al*., 2014; Luo *et al*., 2010; Sarkar *et al*., 2014; Tsuchiya *et al*., 2014; Wang *et al*., 2013, 2014b; Xu *et al*., 2011). To identify whether the UBXN2A/CHIP complex targets mot‐2 in tumor tissues, we decided to examine the presence of the UBXN2A/CHIP/mot‐2 complex in a mouse model of colon cancer. We utilized azoxymethane/dextran sodium sulfate (AOM/DSS) in the C57Bl/6 mouse model (Tanaka *et al*., 2003), as the AOM‐induced tumors accurately mimic the pathophysiological and molecular features of human CRC (Boivin *et al*., 2003; Chen and Huang, 2009; Roy *et al*., 2002). The AOM model develops tumors in the rectum and distal colon, which are the regions of greatest tumor development in humans (De Robertis *et al*., 2011; Thaker *et al*., 2012) (Fig. 8B). After 12 weeks, colons and rectums were dissected. Tumors raised in the colons and rectums, and adjacent normal tissues were isolated on ice‐cold PBS. The collected tissues were homogenized using a lysate buffer. Tissues lysates were subjected to IP using an anti‐UBXN2A antibody (Fig. 8C). WB analysis revealed the presence of mot‐2 pulled down in the presence of UBXN2A in both normal and tumor tissues, and more mot‐2 proteins bound to UBXN2A were observed in tumor lysates dissected from colon and rectum areas as compared to their adjacent normal tissues (lane IV versus II and V versus III). An anti‐CHIP antibody showed the presence of CHIP along with mot‐2 proteins in all UBXN2A lanes (II‐V). However, the level of CHIP proteins showed an increase in tumors originated and developed in the colon section. The existence of a multiprotein complex containing UBXN2A, CHIP, and mot‐2 in tumors raised in colon sections suggests that UBXN2A is a key factor when the CHIP E3 ligase targets mot‐2 for ubiquitination in tumors. While it is beyond the scope of this current study, future investigation utilizing our available colon‐specific knockout (KO) mouse models of CHIP and UBXN2A will determine whether the coexistence of UBXN2A and CHIP is necessary and sufficient to slow tumor growth via suppression of mot‐2 and its downstream tumorigenic pathways in a mouse model of CRC.

### 3.11 UBXN2A haploinsufficient mouse increases mot‐2 protein level, and this phenotype is restricted to colon tissues

To confirm the indispensable role of UBXN2A as the key negative regulator of mot‐2, we utilized a heterozygous mouse model of UBXN2A (Fig. 8D). Mice heterozygous for UBXN2A appear normal and fertile. Dissected tissues from UBXN2A heterozygous mice (UBXN2A^+/−^) showed that haploinsufficient mice express approximately half the levels of UBXN2A compared with their WT littermates (Fig. 8, Panel E). More interestingly, WB revealed the level of mot‐2 protein increases in UBXN2A^+/−^ tissues, particularly ~ twofold increase in the distal colon, while mot‐2 bands did not change markedly in the prefrontal cortex (PFC‐Fig. 8F). Nitrocellulose membranes were additionally probed with anti‐p97 antibody, a known partner of UBXN2A at the ER compartment. Results showed the reduction in UBXN2A leads to reduction in p97 protein levels. Collectively, these *in vivo* results suggest that UBXN2A has a significant physiological effect on mot‐2's turnover in colon tissues. Our ongoing investigation will reveal how UBXN2A, CHIP, and mot‐2 cross talk in CRC patient tissues, including adenoma, adenocarcinoma, and normal intestinal tissues.

## 4 Discussion

Mot‐2 is a potent oncoprotein that is upregulated in many cancers, including human colorectal cancer (Black and Rezvani, 2016; Dundas *et al*., 2005; Gestl and Anne Bottger, 2012; Pilzer *et al*., 2010; Rozenberg *et al*., 2013). Although many studies have elucidated how mot‐2 contributes to tumorigenesis as well as tumor invasion and metastasis, little is known regarding its mechanisms of regulation. Through an unbiased proteomics screen, we identified CHIP as a potential E3 ubiquitin ligase and negative regulator of mot‐2. We found that UBXN2A, a ubiquitin‐like protein, promotes CHIP‐dependent polyubiquitination and downregulation of mot‐2 by the 26S proteasome (Fig. 8G). In addition to its independent functions, UBXN2A is a cofactor of p97 (CDC48) protein. The p97 protein can target cytoplasmic proteins for degradation tagged by different members of the UBXD family (Alexandru *et al*., 2008). Further studies are required to show whether UBXN2A binds to mot‐2 to sequentially promote mot‐2's ubiquitination and proteasomal degradation by CHIP and p97, respectively.

The combination of evidence and the results illustrated in Figs 2D and 3F as well as Fig. S2 indicates a sequence of events following UBXN2A induction. These events start with apoptosis and G1 arrest in cells followed by G2/M arrest after 72 h. However, the reduced migration of cells expressing GFP‐UBXN2A shown in Fig. 3F indicates expression of GFP‐UBXN2A can additionally target other tumorigenic functions mediated by mot‐2 in cancer cells. Overexpression of mot‐2 contributes to the migration pathway in cancer cells (Na *et al*., 2016). Inhibition of cell proliferation and pro‐apoptotic functions of UBXN2A does not rule out UBXN2A's antimigration function. An example for this scenario is flubendazole, which inhibits cell proliferation by G2/M cell cycle arrest and pro‐apoptosis but has no effect on cell migration (Zhou *et al*., 2018). This example supports the notion that induction of apoptosis and cell cycle arrest is not always sufficient to be an antimigratory factor as well. In sum, the results from our study demonstrate that UBXN2A appears to exert antiproliferation and pro‐apoptosis as well as antimigration effects in colon cancer cells. These actions of UBXN2A in cancer cells are likely mediated through mot‐2 signaling pathways, while we cannot eliminate mot‐2 independent pathways. Further studies are necessary to examine these individual mechanisms.

Most significantly, we found that induction of UBXN2A or enhancing its expression with plant alkaloid VTD can destabilize mot‐2, resulting in strong inhibitory effects on mot‐2's downstream effectors in tumorigenesis and cell migration. Our gradient ultracentrifugation showed overexpression of UBXN2A leads to downregulation of mot‐2 and its chaperone partner HSP60 in both cytoplasm and mitochondrial compartments. VTD‐dependent downregulation of mot‐2/HSP60 in cancer cells could be one of the molecular mechanisms behind the VTD‐induced apoptosis and growth arrest. It is noteworthy to say that Burbulla *et al*. have shown HSP60 proteins remain intact or increase after mortalin reduction or knockdown in human fibroblasts and neuronal cells, respectively (Burbulla *et al*., 2014; Wadhwa *et al*., 2005), suggesting two distinct regulatory mechanisms between noncancerous cells and colon cancer cells. Similarly, we observed p97 protein levels decrease in UBXN2A^+/−^ mice in examined tissues. As a partner of p97, it is possible that the reduction in UBXN2A protein in the haploinsufficient mouse alters stability of p97 or interferes with modification and/or hexamer formation. Additional research will be needed to further investigate the relation between UBXN2A and p97 at the ER level in both *in vitro* and *in vivo* models.

Our ubiquitination assays combined with rescue experiments in Fig. 5 indicate UBXN2A may enhance CHIP's substrate ubiquitin ligase activity to polyubiquitinate mot‐2. A similar enhancement mechanism was explained for CHIP E3 ubiquitin ligase activity against oncoproteins in prostate and lung cancers (Sarkar *et al*., 2014; Yoo *et al*., 2018). Our results suggest that CHIP alone is unable to function as a potent E3 ligase against mot‐2, probably due to its low levels and relatively weak self‐association in physiological settings. In contrast, the UBXN2A/CHIP association may occur more readily, which triggers the formation of an active form of CHIP E3 ligase to processively polyubiquitinate mot‐2.

Our finding that the UBXN2A/CHIP complex is the first negative regulator of mot‐2 has several important translational implications. First, because mot‐2 is a central player in both the p53 pathway (Gestl and Anne Bottger, 2012) and epithelial–mesenchymal transition (EMT) (Na *et al*., 2016) and it can simultaneously affect various biological processes including tumorigenesis, invasion, and metastasis, this current study is unique in presenting a potential therapeutic role for UBXN2A/CHIP in the regulation of some or all of the above fundamental biological events altered by mot‐2 in cancer cells. Second, because mot‐2 shows overexpression in a wide variety of human cancer cells, VTD‐dependent downregulation of mot‐2 at the protein level through CHIP‐medicated ubiquitination may have a unique translational value. Due to the important roles of mot‐2 in cancer migration and invasion (Chen *et al*., 2014; Na *et al*., 2016; Ryu *et al*., 2014; Yi *et al*., 2008), establishing VTD as a potent mot‐2 destabilizer molecule will set up a unique platform for new preclinical drug development in different types of cancer, including colorectal cancer. Clearly, more work will be required to determine how UBXN2A facilitates/mediates CHIP‐dependent ubiquitination and proteasomal degradation of mot‐2 oncoprotein *in vitro* and *in vivo*. Ongoing experiments in our laboratory are using truncated and mutant forms of UBXN2A and CHIP proteins to enhance our understanding of UBXN2A/CHIP functions in cancer cells and mouse models of colon cancer. In addition, future studies in our group will determine the lysine residues on human mot‐2 that are ubiquitinated by CHIP using tandem mass spectrometry as previously described for HSP70 and HSP90 (Kundrat and Regan, 2010).

Finally, although our study demonstrated a role for UBXN2A in negative regulation of mot‐2 via the CHIP E3 ubiquitin ligase, it is possible that the UBXN2A/CHIP complex may also negatively regulate other oncoproteins or their tumorigenic pathways activated in both primary and metastatic tumors. Current evidence indicates that the reduction in CHIP expression promotes tumor growth in selected types of cancer, including colorectal cancer (Blessing *et al*., 2014; Liu *et al*., 2015; Wang *et al*., 2013, 2014b). Therefore, it will be very interesting to further explore whether UBXN2A can reverse cancer cell growth and invasiveness in tumors with a low level of CHIP.

## 5 Conclusions

Our study has identified UBXN2A/CHIP as a unique negative regulator of mot‐2 oncoprotein. We demonstrate that UBXN2A, as a ubiquitin‐like protein, facilitates mot‐2's ubiquitination by the CHIP E3 ubiquitin ligase in both *in vitro* and *in vivo* models. Selective destabilization of mot‐2 protein by UBXN2A leads to inhibition of cell proliferation and decreased cancer cell migration. Further understanding their functional interactions in both mice and human tumor samples will have significant impact on the diagnosis, prognosis, and therapy of human tumors with a high level of mot‐2. Finally, establishing VTD, a UBXN2A enhancer molecule, as an antimigration agent will set up a unique platform for new preclinical drug development in colon cancer research.

## Author contributions

SS and AH carried out the experiments. RS performed *in vitro* ubiquitination assays. DM conducted ultracentrifuge gradient experiments. JIS, CJN, ADH, and TK carried out confocal microscopy studies. XW advised on cell biology and concept of designed experiments as well as provided the CHIP knockout mouse model. YW and JY performed the orthogonal ubiquitin transfer (OUT) technique and provided reagents plus CHIP knockout cell lines. JY provided critical feedback and helped shape the research, analysis, and manuscript. KR designed experiments, analyzed data, and wrote the paper. All authors have reviewed and approved the manuscript for submission.

## Supporting information


**Fig. S1.** UBXN2A/CHIP regulates stability of mot‐2 oncoprotein.

**Fig. S2.** UBXN2A arrests G1 then G2/M cell cycles in a sequential manner.

**Fig. S3.** VTD destabilizes mot‐2 via upregulation of UBXN2A.

**Fig. S4.** VTD decreases mot‐2 protein levels in LoVo cells, a well‐differentiated colon cancer cell line.

**Fig. S5.** VTD decreases mot‐2 protein levels in HCT‐116 cells, a poorly‐differentiated colon cancer cell line.

**Fig. S6.** VTD regulates mot‐2 in HepG2 (liver) and MCF‐7 (breast) cancer cells.

**Fig. S7.** Ubiquitinated mot‐2 are present in colon tissues, particularly in the distal colon.

**Table S1.** Antibodies, manufacturers, and their dilutions used for WBs.

**Table S2.** The orthogonal UB transfer (OUT) screen shows mot‐2 is a substrate for the CHIP E3 ubiquitin ligase.
