# Supplementary material for: UBXN2A enhances CHIP‐mediated proteasomal degradation of oncoprotein mortalin‐2 in cancer cells
Source: Mol Oncol. 2018 Sep 3;12(10):1753–77. doi: 10.1002/1878-0261.12372 (PMC6166003; doi:10.1002/1878-0261.12372)
Supplement: Supplementary file 8 — Table S1. Antibodies, manufacturers, and their dilutions used for WBs. [file MOL2-12-1753-s008.docx]

| **Name** | **Manufacturer** | **Dilution for WB** |
| --- | --- | --- |
| Rabbit polyclonal anti-UBXN2A against #C-IQRLQKTASFRELS peptide located in the c-terminus of human UBXN2A (#NM_181713) | Pacific Immunology Corp | 1:1000 |
| Anti-CHIP (C-terminal) antibody | Sigma | 1:1000 |
| CHIP (C3B6) | Cell signaling | 1:1000 |
| Anti-GRP75 (mot-2) antibody (D-9) | Santa Cruz biotechnology | 1:2000 |
| Anti-ubiquitin antibody (FK2) | Enzo | 1:1000 |
| Anti-NSFL1C antibody (p47) | ABCAM | 1:1000 |
| Anti-HSC70 | Santa Cruz biotechnology | 1:5000 |
| Anti-HSP60 | Santa Cruz biotechnology | 1:2000 |
| Anti- VCP (D-9)/p97 | Santa Cruz biotechnology | 1:2000 |
| Anti-β-actin | Sigma | 1:5000 |
| Mouse anti-Glyceraldehyde-3-Phosphate Dehydrogenase antibodies (anti-GAPDH, loading controls and cytoplasmic marker). | Millipore | 1:20000 |
| IRDye 800CW Goat anti-Rabbit IgG (H+L), | LI-COR Corporate | 1:3000 |
| IRDye 800CW Goat anti-Mouse IgG (H+L), | LI-COR Corporate | 1:3000 |

Table S1: Details of antibodies, manufacturers and the dilution used for WBs.
