# Supplementary material for: UBXN2A enhances CHIP‐mediated proteasomal degradation of oncoprotein mortalin‐2 in cancer cells
Source: Mol Oncol. 2018 Sep 3;12(10):1753–77. doi: 10.1002/1878-0261.12372 (PMC6166003; doi:10.1002/1878-0261.12372)
Supplement: Supplementary file 9 — Table S2. The orthogonal UB transfer (OUT) screen shows mot‐2 is a substrate for the CHIP E3 ubiquitin ligase. [file MOL2-12-1753-s009.docx]

| **Description** | **Gene** | **Accession** | **CalcPI** | **#AA** | **MW[kDA]** |
| --- | --- | --- | --- | --- | --- |
| Stress-70 protein, mitochondrial OS=Homo sapiens GN=HSPA9 PE=1 SV=2 | Mot-2, HspA9 | P38646 | 6.16 | 679 | 73.635 |
|  |  | **Control 1** |  |  |  |
| Coverage | #Peptide | #Unique Peptides | Area | #PSMs |  |
| 19.44 | 11 | 11 | 5.10E+07 | 16 |  |
|  |  | **Control 2** |  |  |  |
| Coverage | #Peptide | #Unique Peptides | Area | #PSMs |  |
| 0 | 0 | 0 | 0 | 0 |  |
|  |  | **Control 3** |  |  |  |
| Coverage | #Peptide | #Unique Peptides | Area | #PSMs |  |
| 1.62 | 1 | 1 | 949076.125 | 1 |  |

Table S2: The orthogonal UB transfer (OUT) screen shows mot-2 is a substrate for the CHIP E3 ubiquitin ligase.

|  |  | **OUT Screen 1** |  |  |  | LOG2 (PSM Ratio)  CHIP/CTL Screen 1  0.64 |
| --- | --- | --- | --- | --- | --- | --- |
| Coverage | #Peptide | #Unique Peptides | Area | #PSMs |  |  |
| 32.25 | 18 | 18 | 140000000 | 25 |  |  |
|  |  |  |  |  |  |  |
|  |  | **OUT Screen 2** |  |  |  |  |
| Coverage | #Peptide | #Unique Peptides | Area | #PSMs |  | CHIP/CTL Screen 2  6.64 |
| 11.49 | 6 | 6 | 36000000 | 7 |  |  |
|  |  |  |  |  |  |  |
|  |  | **OUT Screen 3** |  |  |  |  |
| Coverage | #Peptide | #Unique Peptides | Area | #PSMs |  | CHIP/CTL Screen 3  3.58 |
| 15.91 | 9 | 9 | 28597752.34 | 12 |  |  |
