# Supplementary material for: UBXN2A enhances CHIP‐mediated proteasomal degradation of oncoprotein mortalin‐2 in cancer cells
Source: Mol Oncol. 2018 Sep 3;12(10):1753–77. doi: 10.1002/1878-0261.12372 (PMC6166003; doi:10.1002/1878-0261.12372)
Supplement: Supplementary file 10 [file MOL2-12-1753-s010.docx]

**Supplementary Figure legends**

**Supplementary Figure 1. Stability of the mot-2 oncoprotein is regulated by the UBXN2A/CHIP pathway in cancer cells. Panel A**. To verify CHIP is important in the UBXN2A-CHIP-mot-2 complex, colon tissue (LI) lysates from C57Bl/6 WT (CHIP +/+) or CHIP knockout (CHIP-/-) were subjected to IP using anti-UBXN2A antibodies immobilized on magnetic IgA. WB showed mot-2 protein can be pulled down particularly from WT colon lysates where CHIP proteins are present. **Panel B**. HEK293 cells stably expressing scramble shRNA or sRNA against CHIP were treated with DMSO, emetine, or combination of emetine and bortezomib for 24 hours. Cell lysates were subjected to WB experiments using anti-mot-2, anti-CHIP, and anti-GAPDH antibodies. Results indicate that CHIP dominantly contributes to the stability of mot-2 proteins. **Panel C**. U2OS stably expressing scramble shRNA or shRNA against UBXN2A (clone 42 and 92) were subjected to WB experiments using anti-UBXN2A, anti-mot-2, anti-HSC70 and anti-GAPDH (loading control) antibodies. Experiments illustrated in Panel B and C were repeated two times with similar results.

**Supplementary Figure 2. UBXN2A causes G1 then G2/M cell cycle arrest in a sequential manner. Panels A and B.** Cell cycle analysis was performed using propidium iodide (PI). Inducible HCT-116 cells were treated with DOX for periods of up to 96 hours, and DNA content was examined by PI staining and flow cytometry, with the histograms shown in Panels A (GFP-empty) and B (GFP-UBXN2A). Quantitation of the data in **Panel C** show cells expressing GFP-empty had DNA content reflecting distribution in G1, S, or G2 and/or M phases as expected with no major changes between hours 0 and 96. However, **Panel D** shows induction of UBXN2A causes G1 arrest, particularly after 48 hours, which is followed by a progressive increase of G2/M-phase cell population until 96 hours. These experiments were conducted in triplicate. Graph Pad Prism was used to determine the percentage of cells in each cell cycle phase.

**Supplementary Figure 3. VTD-dependent degradation of mot-2 is mediated by UBX2A. Panel A.** Re-probing of the nitrocellulose membrane illustrated in Figure 5C with anti-ubiquitin (FK2 clone) showed shRNAs against CHIP and UBXN2A plus re-expression of GFP-UBXN2A have no major effect on total ubiquitinated proteins in HCT-116 cells. **Panel B**. LoVo cells were treated with different concentrations of VTD for 72 hours. Cell lysates were subjected to WB. **Panel C** shows VTD significantly increases UBXN2A proteins in a dose-dependent manner in LoVo colon cancer cells (n=3, *P<0.05, **P<0.01 and ***P<0.001- Tukey’s multiple comparison test, mean ± SE). **Panel D**. HEK293 cells stably expressing shRNA against CHIP were transfected with shRNAs against UBXN2A (Clones 42 and 92) or a scramble shRNA. After 48 hours, cells were treated with VTD (100μM) for another 72 hours. WB analysis shows VTD failed to decrease the protein level of mot-2 in the absence of UBXN2A and CHIP (lane III versus II and VI versus V).

**Supplementary Figure 4. VTD significantly decreases mot-2 expression in LoVo colon cancer cells**. **Panel A**. LoVo cells were treated with respective concentrations of VTD or vehicle (DMSO) for 72 hours. Following staining with Alexa Fluor 546 anti-mot-2 (Red) and DAPI (Blue), cells were imaged using confocal microscopy. Data was normalized with DMSO. **Panel B**. Quantitative analysis of mot-2 levels from IF images was performed using Image-J software (n=150, ***P<0.001- Tukey’s multiple comparison test, mean ± SE). Cells were analyzed by confocal microscope and photographed at 60x magnification.

**Supplementary Figure 5. VTD significantly decreases mot-2 expression in HCT-116 colon cancer cells in a dose-dependent manner.** HCT-116 cells were treated with different concentrations of VTD for 72 hours. **Panels A-B**. Flow-cytometry analysis shows VTD significantly decreases mot-2 protein in cells in a dose-dependent manner. These results show VTD-dependent destabilization of mot-2 in HCT-116 poorly differentiated cells can efficiently start at lower doses in comparison to LoVo, a well-differentiated cell line (main Fig. 6). **Panels C-D**. HCT-116 cells treated with VTD for 72 hours were additionally subjected to WB. Measurement of signals further confirmed that VTD significantly reduces mot-2 proteins in a dose-dependent manner in HCT-116 cells (n=3, *P<0.05, **P<0.01 and ***P<0.001- Tukey’s multiple comparison test, mean ± SE).

**Supplementary Figure 6. VTD differentially decreases mot-2 expression in HepG2 and MCF-7 cells. Panels A and C.** Cell lines HepG2, a human liver cancer, and MCF-7, a human breast cancer, were treated with different concentrations of VTD for 72 hours. Cells were fixed and stained with anti-mot-2 antibodies as well as DAPI. **Panels B and D**. Quantitative analysis of mot-2 (red signals) from IF images was performed using Image-J software (n=150, ***P<0.001- Tukey’s multiple comparison test, mean ± SE). The IF results in four different cell lines (LoVo, HCT-116, HepG2, and MCF-7) suggest that VTD can decrease the protein levels of mot-2 in a cell context-dependent manner. Cells were analyzed by confocal microscope and photographed at 60x magnification.

**Supplementary Figure 7. Ubiquitinated forms of mot-2 are found in colon tissues, particularly in the distal colon. Panels A and B**. Colon tissues were dissected from two C57Bl/6 mice (M1 and M2). The proximal section was determined and separated from the distal section in an ice-cold PBS plate. Total protein concentrations were determined, and equal amount of proteins were incubated with anti-ubiquitin antibodies (clone FK2) immobilized on IgG magnetic beads for 2 hours at 4°C with gentle rocking. Pulled-down ubiquitinated proteins were subjected to WB. Panel A. In contrast to *in vitro* results, ubiquitinated mot-2 appeared in the form of only one or two clear bands above the typical mot-2 molecular weight. Panel B. The nitrocellulose membrane used in Panel A was re-probed by the clone FK2 anti-ubiquitin antibody. The ubiquitinated proteins displayed a typical “ladder” of bands which were highly similar in both proximal and distal colon sections. These *in vivo* results suggest that mot-2 is indeed ubiquitinated in colon tissues. However, the ubiquitinated forms of mot-2 are slightly different between the proximal and distal sections, indicating the involvement of diverse underlying mechanisms.

Supplementary Table 1: **Details of antibodies, manufacturers and the dilution used for WBs**. Dilution of antibodies for other experiments including flow-cytometry and IF can be found in main text.

Supplementary Table 2: **Mot-2 as a CHIP substrates identified by orthogonal UB transfer (OUT).** Numbers of peptide-spectrum match (PSM) for xUB-conjugated proteins purified from cells expressing the full length OUT cascade of CHIP (xUba1-xUbcH5b-xCHIP) are listed under "OUT Screen 1", "OUT Screen 2" and "OUT Screen 3", respectively. Numbers of PSM for xUB-conjugated proteins purified from control cells expressing the truncated OUT cascade (xUba1-xUbcH5b) without xCHIP are listed under "Control 1", "Control 2" and "Control 3", respectively. The OUT screens were repeated three times, each time with different preparation of the cells. Log2 values of the PSM ratio of mot-2 between cells expressing the OUT cascade of CHIP and control cells expressing the xUba1-xUbcH5b pair are listed.
